# Adaptive Bayesian credible bands in regression with a Gaussian process prior

**DOI:** 10.1007/s13171-019-00185-0

**Published:** 2019-11-15

**Authors:** Suzanne Sniekers, Aad van der Vaart

**Affiliations:** Mathematical Institute, Leiden University, P.O. Box 9512, 2300 RA Leiden, The Netherlands

**Keywords:** Credible band, Coverage, Uncertainty quantification, Nonparametric Bayes, 62G15, 62G05, 62G20

## Abstract

A credible band is the set of all functions between a lower and an upper bound that are constructed so that the set has prescribed mass under the posterior distribution. In a Bayesian analysis such a band is used to quantify the remaining uncertainty on the unknown function in a similar manner as a confidence band. We investigate the validity of a credible band in the nonparametric regression model with the prior distribution on the function given by a Gaussian process. We show that there are many true regression functions for which the credible band has the correct order of magnitude to be used as a confidence set. We also exhibit functions for which the credible band is misleading.

## Introduction and main results

Suppose that we observe a vector **Y**_*n*_ := (*Y*_1,*n*_,…,*Y*_*n*,*n*_)^*T*^ with coordinates distributed according to

1.1 $$  Y_{i,n} = f(x_{i,n}) + \varepsilon_{i,n}, \qquad i\in \{1,\ldots, n\}. $$

Here the parameter is a function $f: [0,1] \to \mathbb {R}$, the design points (*x*_*i*,*n*_) are a known sequence of points in [0,1], and the (unobservable) errors *ε*_*i*,*n*_ are independent standard normal random variables. In this paper we investigate a nonparametric Bayesian method to estimate the regression function *f*, based on a Gaussian process prior. We are interested in the usefulness of the resulting posterior distribution for quantifying the remaining uncertainty about the function. More precisely, the posterior distribution allows to construct a *credible band*: a ball for the (weighted) uniform norm around the posterior mean of prescribed posterior probability. We investigate to what extent such a band has similar properties as a (frequentist) confidence band.

The performance of a credible band depends strongly on the combination of prior and true regression function. Furthermore, for priors that “adapt” to the true function through a regularity parameter it depends on the method of adaptation. In this paper we illustrate this with a case study of a special prior, namely scaled Brownian motion. For simplicity we choose the design points *x*_*i*,*n*_ to be equally spaced and equal to *x*_*i*,*n*_ = *i*/*n*_+_, where *n*_+_ = *n* + 1/2. For *W* = (*W*_*t*_,*t* ∈ [0,1]) a standard Brownian motion we take $\sqrt c W$ as a prior for *f*, where the scaling parameter *c* > 0 will be set by an empirical or hierarchical Bayes method. Our insistence on a band rather than a ball for the *L*_2_-norm distinguishes this paper from earlier work, such as Szabó et al. (2015a) and Sniekers and van der Vaart (2015a). From the point of view of visualisation bands are preferable for data-analysis.

In the Bayesian setup the observations are distributed according to the model, where *W* and the errors *ε*_*i*,*n*_ are independent,

1.2 $$  Y_{i,n} = \sqrt{c} W_{x_{i,n}} + \varepsilon_{i,n}, \qquad i\in \{1,\ldots, n\}. $$

By definition the posterior distribution of *f* given **Y**_*n*_ and *c* is the conditional distribution of $f=\sqrt c W$ given **Y**_*n*_ and *c* in this model; we denote it by π_*n*_(⋅|**Y**_*n*_,*c*). By standard properties of Gaussian distributions this can be seen to be the distribution of a Gaussian process. It has a version with continuous sample paths and we can consider the posterior distribution formally as a Borel law on *C*[0,1]. We denote its mean and covariance function by

1.3 $$ \begin{array}{@{}rcl@{}} \hat f_{n,c}(x)&=&\mathord\mathrm{E} \left( f(x) | \mathbf{Y}_{n},c\right), \end{array} $$

1.4 $$ \begin{array}{@{}rcl@{}} \sigma_{n}(x,y,c)&=&\text{cov}\left( f(x), f(y) | \mathbf{Y}_{n},c\right). \end{array} $$

It is natural to center a credible set for the function *f* at the posterior mean. We shall see that the posterior variance *σ*_*n*_(*x*,*x*,*c*) hardly depends on *x*, so that equal width intervals for different *x* are natural, yielding a band if applied simultaneously. We shall consider a *credible band* of the form:

1.5 $$ C_{n} (c,L)= \left\{f: \|f- \hat f_{n,c}\|_{\infty}< L w_{n}(c)\right\}, $$

where $\|f\|_{\infty }=\sup _{x\in J_{n}}|f(x)|$ is the uniform norm over the interval *J*_*n*_ = [1/*l*_*n*_,1 − 1/*l*_*n*_], where $l_{n}\rightarrow \infty $ is a fixed sequence with $l_{n}\ll \sqrt {\log n}/\text {loglog} n$, *L* is a constant, and *w*_*n*_(*c*) is a posterior quantile of the uniform norm of $f-\hat f_{n,c}$: for some *η* ∈ (0,1),

1.6 $$ {\Pi}_{n}\left( \|f-\hat f_{n,c}\|_{\infty}< w_{n}(c) | \mathbf{Y}_{n}, c\right)=\eta. $$

We restrict to the subinterval *J*_*n*_ ⊂ [0,1] to avoid boundary effects of the Brownian prior, and have inserted a constant *L* in definition Eq. 1.5 in order to make up for a possible discrepancy between a Bayesian credible level and frequentist confidence level. The credible level *η* will be fixed throughout. For practice we recommend to use the value *L* = 1, since this corresponds to the true Bayesian procedure. It will be apparent from our results that frequentist coverage of a given parameter *f* is only ensured if *L* is large enough relative to parameters such as *𝜖* in Eq. 1.13. There is no way to estimate this from the data, and we can only be assured that the *order* of magnitude of the Bayesian band is also correct in the frequentist sense.

That the distribution of the observations **Y**_*n*_ depends on *f* only through its values at the design points *x*_*i*,*n*_ motivates to consider also “discrete bands”, where the argument *x* is restricted to the design points, of the form

1.7 $$ {C_{n}^{d}}(c,L) = \left\{f: \max_{i\in\mathcal{J}_{n}}|f(x_{i,n})- \hat f_{n,c}(x_{i,n})|< L {w_{n}^{d}}(c)\right\}, $$

where $\mathcal {J}_{n}=\{i: x_{i,n}\in J_{n}\}$ and ${w_{n}^{d}}(c)$ is determined so that

1.8 $$  {\Pi}_{n}\left( \max_{i\in\mathcal{J}_{n}}|f(x_{i,n})-\hat f_{n,c}(x_{i,n})|< {w_{n}^{d}}(c) | \mathbf{Y}_{n}, c\right)=\eta. $$

As the design points form a grid with mesh width of the order 1/*n*, one may expect the bands Eqs. 1.5 and 1.7 not to differ much, but the difference depends both on the prior and the true function *f*.

It will be shown below that the widths of the bands satisfy

$$ w_{n}(c)\asymp {w_{n}^{d}}(c)\asymp\sqrt{\log (cn)}\left( \frac cn\right)^{1/4}. $$

Here the logarithmic factor arises because of the uniform norm, while the factor (*c*/*n*)^1/4^ gives the order of magnitude of the posterior standard deviation *σ*_*n*_(*x*,*x*,*c*)^1/2^ of *f*(*x*). This shows that for fixed *c* the band will never be narrower than *n*^− 1/4^, which is disappointing if *f* is smooth. It also suggests that for fixed *c* the band will not cover if *f* is too rough, as one cannot expect to estimate a very rough function at nearly *n*^− 1/4^ precision. In practice one tries to overcome these problems by choosing a suitable value of *c* from the data. Two standard methods are, for $I_n=[(\log n)/n, n/\log n]$,

1.9 $$  \hat c_{n}=\text{argmin}_{c\in I_n} \left[\log \det{\Sigma}_{n,c}+\mathbf{Y_{n}^{T}} {\Sigma}_{n,c}^{-1}\mathbf Y_{n}\right] $$

and

1.10 $$  \hat c_{n} =\text{argmin}_{c\in I_n}\left[\text{tr}\left( (I-{\Sigma}_{n,c}^{-1})^{2}\right)-\text{tr}({\Sigma}_{n,c}^{-2})+\mathbf{Y_{n}^{T}}{\Sigma}_{n,c}^{-2}\mathbf Y_{n}\right]. $$

Here Σ_*n*,*c*_ = *I* + *cU*_*n*_, where *U*_*n*_ is the *n* × *n* covariance matrix of standard Brownian motion at the design points: (*U*_*n*_)_*i*,*j*_ = *x*_*i*,*n*_ ∧ *x*_*j*,*n*_. The first method (1.9) is exactly the maximum likelihood estimator of *c* based on the Bayesian marginal distribution of **Y**_*n*_: the distribution of the right side of Eq. 1.2, with *c* viewed as the only unknown parameter. The second method corresponds to minimizing an unbiased estimate of the quadratic risk function of the estimator $\left (\hat f_{n,c}(x_{1,n}),\ldots , \hat f_{n,c} (x_{n,n})\right )$, and goes back to the literature on penalized estimation. See Wahba (1983) and Sniekers and van der Vaart (2015a) for further discussion. Given either of these estimators one may construct a credible band by simply substituting $\hat c_{n}$ in Eq. 1.5 or Eq. 1.7.

An alternative to these empirical Bayes methods is the hierarchical Bayes method, which equips *c* with a (hyper)prior. Following Sniekers and van der Vaart (2015a) we shall take an inverse gamma prior truncated to the interval *I*_*n*_: the prior density of *c* satisfies, for some fixed *κ*,*λ* > 0,

1.11 $$  \pi(c)\propto c^{-1-\kappa} e^{-\lambda/c}, \qquad c\in I_{n}. $$

This leads to a posterior distribution for *c*, from which we may extract two nontrivial quantiles $\hat c_{1,n}$ and $\hat c_{2,n}$ and next use the credible set $\cup _{\hat {c}_{1,n}<c<\hat {c}_{2,n}}$*C*_*n*_(*c*,*L*) or $\cup _{\hat {c}_{1,n}<c<\hat {c}_{2,n}} {C_{n}^{d}}(c,L)$. It was shown in Sniekers and van der Vaart (2015a) that the hierarchical Bayes method is closely linked to the likelihood-based empirical Bayes method Eq. 1.9 in that the posterior distribution of *c* will concentrate near the likelihood-based empirical Bayes estimator $\hat c_{n}$.

One can devise methods to adapt the scaling parameter *c* to the data that are targeted especially to the uniform norm (see Yoo and van der Vaart 2018), but we shall not consider them in this paper. Our interest here is to stick strictly to the Bayesian paradigm, as best embraced by the hierarchical Bayes and likelihood-based empirical Bayes methods. We then ask for which true regression functions *f* the resulting credible bands work and for which not.

It was shown in Low (1997), Juditsky and Lambert-Lacroix (2003), Cai and Low (2004), Cai and Low (2006), Robins and van der Vaart (2006), and Hoffmann and Nickl (2011) that nonparametric adaptive confidence sets can only be honest (i.e. possess coverage uniformly in the parameter *f* ) if the true regression function possesses special properties. Bayesian credible sets can of course not beat this fundamental limitation, and hence we need to impose conditions on the true regression function. For deterministic *c* = *c*_*n*_ it is enough that the prior is not smoother than the true regression function (i.e. *c* is not too small). This case was discussed in Sniekers and van der Vaart (2015b) (and Knapik et al. (2011)) for credible intervals, but the results generalize to bands. The finding can be understood as a consequence of the bias variance trade-off: a smooth prior will make the band narrow (small variance), but give a large bias on a rough true function; if these are not traded off properly, then coverage fails. In the present paper we consider the more interesting and more complicated case of a data-based choice of *c*. In this case coverage will hold only if the true *f* satisfies additional conditions that prevent $\hat c_{n}$, or the location of the posterior distribution of *c*, to be too small, which would make the bias bigger than the posterior spread. We consider two types of such conditions: first a combination of self-similarity and a Hölder condition, and second functions *f* characterized as realizations from a prior.

The first type of assumption is in terms of the eigenbasis of Brownian motion, given by

1.12 $$  e_{j}(x) = \sqrt{2} \sin\left[\left( j-\tfrac{1}{2}\right)\pi x\right],\qquad j=1,2,\ldots. $$

We call a function *f* *self-similar* of order *β* > 0 if its sequence of Fourier coefficients (*f*_*j*_) with respect to this basis satisfies, for some positive constants *M*,*ρ*,*ε* and every *m*,

1.13 $$  \sup_{j\ge 1}j^{1/2+\beta}|f_{j}|\le M,\qquad\text{ and }\qquad \sum\limits_{j=m}^{\rho m} {f_{j}^{2}}\ge \varepsilon\frac{M^{2}}{m^{2\beta}}. $$

Functions such that *f*_*j*_ ≍ *j*^− 1/2−*β*^ are simple examples. The condition is the same as in Szabo et al. (2015a, 2015b) and similar to conditions introduced in Hoffmann and Nickl (2011), Giné and Nickl (2010), Bull (2012), and Bull and Nickl (2013). It requires that the “total energy” in every sufficiently large block of “frequencies” is at least a fraction of the “total possible energy” in the signal.

Let $\mathcal {F}_{\alpha ,\beta ,\varepsilon ,\rho }$ be the set of all functions that are self-similar of order *β* for given *ε* and *ρ* and some *M* and possess Hölder norm of order *α* smaller than *M*: i.e. $\|f\|_{\infty }\le M$ and |*f*(*x*) − *f*(*y*)|≤ *M*|*x* − *y*|^*α*^ if *α* ≤ 1 and $|f^{\prime }(x)-f^{\prime }(y)|\leq M |x-y|^{\alpha -1}$ if *α* ∈ (1,2].

### Theorem 1 (Coverage).

For *C*_*n*_(*c*,*L*) given in Eq. 1.5, let $\hat C_{n}(L)$ be equal to $C_{n}(\hat c_{n},L)$ for $\hat c_{n}$ defined by the likelihood-based or risk-based empirical Bayes method Eqs. 1.9 or 1.10. If $2\geq \alpha \ge \beta >\frac 12$, then for sufficiently large *L*,

$$ \liminf_{n\rightarrow\infty} \inf_{f\in\mathcal{F}_{\alpha,\beta,\varepsilon,\rho}} \Pr_{f}\left( f\in \hat {C}_{n}(L)\right) \ge \eta. $$

The same is true if $\hat C_{n}(L)$ is equal to $\cup _{\hat c_{1,n}<c<\hat c_{2,n}}C_{n}(c,L)$, for $\hat c_{1,n}<\hat c_{2,n}$ satisfying ${\Pi }_{n}(\hat c_{1,n}<c<\hat c_{2,n} | \mathbf Y_{n})=\eta \in (0,1)$ given a prior density satisfying Eq. 11, and *β* < 1.

The proof of the theorem is given in Sections 4 and 6.

The theorem shows that empirical or hierarchical Bayes credible bands cover the true function if the Hölder smoothness *α* of the function *f* is at least the order of self-similarity *β*. As we shall see later in the proof, this is due to the fact that the behaviour of the estimator $\hat {c}_{n}$ is determined by self-similarity, but the bias for the uniform norm by the Hölder exponent. A value *β* > *α* would lead to a choice of $\hat c_{n}$ that corresponds to overestimating the Hölder smoothness of the function, which leads to poor coverage.

For nice functions the self-similarity index *β* is equal to the Hölder smoothness *α*, but the two indices are not related in general. The self-similarity measures the speed of decrease of the Fourier series, and has an *L*_2_ character, whereas the Hölder smoothness refers to the function in the time domain.

The restriction of the self-similarity constant *β* to be bigger than 1/2 is due to the discrete design. It makes it possible to link the infinite sequence (*f*_*j*_) to the coefficients (*f*_*i*,*n*_) of the vectors **f**_*n*_ relative to the discretized eigen basis, defined in Eq. 3.2, below. We define a function *f* to be *discretely self-similar* of order *β* > 0 if for some positive constants *M*,*ρ*,*ε* and every *m* ≤ *n* and *n*,

$$ \sup_{1\le i\le n}i^{1/2+\beta}\frac{|f_{i,n}|}{\sqrt{n_{+}}}\le M, \qquad\text{ and }\qquad \frac1{n_{+}}\sum\limits_{i=m}^{\rho m\wedge n} f_{i,n}^{2}\ge \varepsilon\frac{M^{2}}{m^{2\beta}}. $$

With self-similarity replaced by discrete self-similarity, Theorem 1 is true for *β* > 0. For *β* > 1/2 self-similarity implies discrete self-similarity (Sniekers and van der Vaart, 2015a).

The second set of functions for which we shall show coverage has a Bayesian flavour. According to the prior the function *f* is a multiple of a sample path of Brownian motion. Hence by the Karhunen-Loève theorem the function can be expanded as a multiple of the infinite sum $(\sqrt 2/\pi ){\sum }_{j=1}^{\infty } Z_{j} e_{j}/(j-1/2)$, for i.i.d. standard normal variables *Z*_1_,*Z*_2_,… and *e*_*j*_ given in Eq. 1.12. If the Bayesian paradigm works at all, the credible band should have the correct order of magnitude for “most of the realisations from the prior”. The following theorem shows that this is indeed true. In fact, coverage pertains for almost every realization of any process of the form, for some *γ* > 0, *α* > 0 and $\delta \in \mathbb {R}$,

1.14 $$  \gamma\sum\limits_{j=1}^{\infty} \frac{Z_{j} e_{j}(x)}{(j+\delta)^{1/2+\alpha}},\qquad x\in [0,1], $$

where *Z*_1_,*Z*_2_,… are independent standard normal random variables. The Brownian motion prior corresponds to *α* = 1/2 and *δ* = − 1/2 and $\gamma = \sqrt {2c}/\pi $.

### Theorem 2 (Coverage).

For ${C_{n}^{d}}(c,L)$ given in Eq. 1.7, let $\hat C_{n}(L)$ be equal to ${C_{n}^{d}}(\hat c_{n},L)$ for $\hat c_{n}$ defined by the likelihood-based or risk-based empirical Bayes method Eq. 1.9 or Eq. 1.10. Let *γ* > 0 and $\delta \in \mathbb {R}$ be given constants. For *α* ∈ (0,1) and the likelihood-based method Eq. 1.9, and for *α* ∈ (0,2) and the risk-based method Eq. 1.10, and for almost every realisation *f* of the process Eq. 1.14, we have that for sufficiently large *L*,

$$ \Pr_{f}\left( f \in \hat C_{n}(L)\right)\rightarrow 1. $$

The same is true if $\hat C_{n}(L)$ is equal to $\cup _{\hat c_{1,n}<c<\hat c_{2,n}}{C_{n}^{d}}(c,L)$, for $\hat c_{1,n}<\hat c_{2,n}$ such that ${\Pi }_{n}(\hat c_{1,n}<c<\hat c_{2,n} | \mathbf Y_{n})=\eta \in (0,1)$ given a prior satisfying Eq. 1.11, and *α* < 1.

The proof of the theorem can be found in Sections 5 and 6. It proceeds by showing that the random functions Eq. 1.14 belong with probability one to a deterministic set, for which coverage is guaranteed.

The preceding theorems show that the credible bands cover the true regression function in some generality, and hence justify the use of the posterior distribution as an expression of remaining uncertainty. The following theorem shows that this coverage is not due to these bands being overly wide.

### Theorem 3 (Diameter).

For *β* < 1 the width of the credible band $\hat {C}_{n}(L)$ in Theorem 1 is $O_{P}\left (\sqrt {\log n} n^{-{\beta }/(1+2\beta )}\right )$; for the risk-based empirical Bayes method this is true for *β* < 2. The same is true for the width of the band in Theorem 2 and every *β* < *α* < 2.

The proof of this theorem is incorporated in the proofs of Theorems 1 and 2.

If the credible band covers the true function, then its width gives the rate of estimation by the posterior mean for the (discrete) uniform norm. The minimax rate of estimation for functions that are Hölder smooth of order *α* is known to be of the order $(n/\log n)^{-{\alpha }/{(1+2\alpha )}}$ (see Stone 1982). Thus in the reasonable case that the order of self-similarity *β* is equal to the Hölder smoothness *α*, the width of the credible bands is close to minimax. However, the width is suboptimal up to a logarithmic factor: the factor $(\log n)^{\alpha /(1+2\alpha )}$ in the minimax rate is replaced by $\sqrt {\log n}$ in Theorem 3. This is caused by the fact that the present methods of choosing *c* are linked to the empirical *L*_2_-norm of *f* rather than the uniform norm. This is immediate for the risk-based empirical based method Eq. 1.10, as it is set up to minimize the *L*_2_-risk. It is also true for the likelihood-based empirical Bayes method Eq. 1.9 and the hierarchical Bayes method, due to the fact that the likelihood is linked to the *L*_2_-norm of the values *f*(*x*_*i*,*n*_). It was shown in Sniekers and van der Vaart (2015a) that these methods do choose an optimal value of *c*, but from the point of view of *L*_2_-loss. For a true function that is regular of order *β* in an appropriate *L*_2_-sense they choose a value of *c* that balances squared bias and variance in the form

$$ \left( \frac 1{\hat cn}\right)^{\beta}\asymp \sqrt{\frac {\hat c}n}. $$

This yields a rate of contraction relative to the *L*_2_-norm of $(\hat c/n)^{1/4}=n^{-\beta /(2\beta +1)}$. (For the Brownian motion prior this is limited to *β* ≤ 2. See Ghosal et al. (2000), Ghosal and van der Vaart (2007), van der Vaart and van Zanten (2007), Szabo et al. (2013), and Ghosal and van der Vaart (2017) for derivations of the rate in the Bayesian setup.) For the uniform norm the variance term incurs an extra logarithmic factor, and the correct trade-off would be

$$ \left( \frac 1{\hat c_{\infty} n}\right)^{\beta}\asymp \log n\sqrt{\frac {\hat c_{\infty}}n}, $$

leading to the minimax rate $(\log n/n)^{\beta /(2\beta +1)}$. However, the Bayesian methods of choosing the scaling in the present paper are not informed about the loss function, and make the trade-off dictated by the likelihood or *L*_2_-risk. The smaller *L*_2_-variance term makes that $\hat c\asymp n^{(1-2\beta )/(1+2\beta )}$ is a logarithmic factor bigger than $\hat c_{\infty }$, and leads to the suboptimal rate $\sqrt {\log n} n^{-\beta /(2\beta +1)}$. Since the loss relative to the minimax rate $(\log n/n)^{\beta /(2\beta +1)}$ is only a logarithmic factor, this is not too bothersome. One gains a unified methodology. For the question of coverage that is central to the present paper it is important that the loss occurs in the variance term and not in the bias term. Coverage requires that the bias is not too large relative to the variance: therefore the fact that $\hat c\gg \hat c_{\infty }$ helps for coverage. This explains that the Bayesian methods of the present paper, although linked to the *L*_2_-norm, can still work for uncertainty quantification relative to the uniform norm.

The positive results on the coverage of credible sets evoked in the preceding theorems are surprising in the light of earlier findings on nonparametric credible sets. In particular, in the papers (Cox, 1993; Freedman, 1999; Johnstone, 2010) credible sets are shown to have zero coverage almost surely. This discrepancy is due to considering nonadaptive credible sets for priors that oversmooth the true functions. On the positive side (Wahba, 1983) gave simulations and heuristic arguments that suggested promising results for credible intervals at the design points. The true functions used in these simulations satisfy the conditions imposed in Theorem 1.

The paper is organised as follows. In Section 2 we collect properties of the posterior mean, in particular its bias relative to the uniform distance, and in Section 3 we discuss the behaviour of the empirical Bayes estimators. Next Sections 4 and 5 contain the proofs of the main result in the empirical Bayes case, where in Section 5 a result of independent interest is obtained, and Section 6 gives the proof in the hierarchical case. Section 7 presents a (counterexample) of a true function, and some pictures of bands. For easy reference and completeness a supplement (Sections 8, 10, and 11) contains adaptations and extensions of results from our earlier papers. The proof of a technical lemma related to these results can be found in Section 9, also in the supplement.

Throughout we denote the interval $ [\log n/n,n/\log n]$ by *I*_*n*_ and [1/*l*_*n*_,1 − 1/*l*_*n*_] by *J*_*n*_, where $l_{n}\rightarrow \infty $ is a fixed sequence with $l_{n}\ll \sqrt {\log n}/\text {loglog} n$. The symbol $\lesssim $ is used to denote “less than up to a multiplicative constant that is universal or fixed within the context”.

## Posterior mean, spread and quantiles

The posterior distribution of *f*(*x*) for a given *x* was studied in Sniekers and van der Vaart (2015b). In this section we present versions of a few results from the latter paper that are uniform in *x* and *c*, and we characterise posterior quantiles of the uniform norm of *f* minus its expectation. For easy reference and completeness, proofs that follow the same lines as in Sniekers and van der Vaart (2015b) are given in Section 8 of the supplement.

The posterior mean of *f*(*x*) is the conditional expectation $\hat {f}_{n,c}(x)=\mathord \mathrm {E}(\sqrt c W_{x} | Y_{1,n},\ldots , Y_{n,n})$, and can be written as a linear combination of the observations:

$$ \hat{f}_{n,c}(x)={\sum}_{i=1}^{n} a_{i,n}(x,c) Y_{i,n} = \mathbf{Y}_{n}^{T} \mathbf{a}_{n}(x,c). $$

The vector of coefficients $\mathbf a_{n}(x,c)=(a_{i,n}(x,c))\in \mathbb {R}^{n}$ is characterised concretely in Proposition 15, in Section 8. The coefficients are essentially a sliding exponential filter of bandwidth (*cn*)^− 1/2^: for $|x-x_{i,n}|\lesssim (cn)^{-1/2}$,

2.1 $$  a_{i,n}(x,c)\sim \frac 1{2n}\sqrt{cn} e^{-|x-x_{i,n}|\sqrt{cn}}. $$

The covariance function of the posterior mean can be expressed in the coefficients **a**_*n*_ as:

$$ \begin{array}{@{}rcl@{}} \tau_{n}(x,y,c) = \text{cov}_{f}\left( \hat f_{n,c}(x), \hat f_{n,c}(y)\right) = \mathbf a_{n}(x,c)^{T}\mathbf a_{n}(y,c). \end{array} $$

Normality and orthogonality imply the independence of the residual $\sqrt c W_{x}-\hat f_{n,c}(x)$ and **Y**_*n*_ in the Bayesian setup. Hence the posterior covariance function as in Eq. 1.4 is equal to the unconditional covariance function of the process $\sqrt c W_{x}-\hat {f}_{n,c}(x)=\sqrt {c}W_{x}-\sqrt {c}\mathbf {W_{n}^{T}}\mathbf a_{n}(x,c)-\mathbf {\varepsilon _{n}^{T}}\mathbf a_{n}(x,c)$ and can be written

$$ \begin{array}{@{}rcl@{}} \sigma_{n}(x,y,c) = c\text{cov} \left( W_{x}-\mathbf{W_{n}^{T}}\mathbf a_{n}(x,c), W_{y} - \mathbf{W_{n}^{T}}\mathbf a_{n}(y,c) \right) + \mathbf a_{n}(x,c)^{T}\mathbf a_{n}(y,c). \end{array} $$

For technical reasons we also introduce a slight adaptation of this function, given by

$$ \begin{array}{@{}rcl@{}} \bar \sigma_{n}(x,y,c) = c\text{cov} \left( W_{x} 1^{T}\mathbf a_{n}(x,c) - \mathbf{W_{n}^{T}}\mathbf a_{n}(x,c), W_{y} 1^{T}\mathbf a_{n} - \mathbf{W_{n}^{T}}\mathbf a_{n}(y,c) \right) + \mathbf a_{n}(x,c)^{T}\mathbf a_{n}(y,c). \end{array} $$

The numbers $1^{T}\mathbf a_{n}(x,c)={\sum }_{i=1}^n a_{i,n}(x,c)$ will be seen to be close to 1, so that $\bar \sigma _{n}\approx \sigma _{n}$.

The following lemma lists the most important properties of these quantities; its proof can be found in Section 9.

### Lemma 4.

Fix an arbitrary sequence $\eta _{n}\rightarrow 0$. The approximation Eq. 2.1 is valid uniformly in *i* and *x* and *c* such that $|x-x_{i,n}|\sqrt {cn}<1/(2\eta _{n})$ and *c*/*n* ≤ *η*_*n*_ and $\left (x\wedge (1-x)\right )\sqrt {cn}\ge 1/\eta _{n}$. Furthermore, for every *i* the coefficients *a*_*i*,*n*_(*x*,*c*) are nonnegative, and uniformly in *c*/*n* ≤ *η*_*n*_ and *x* ∈ [1/*n*_+_, 1],

2.2 $$ \begin{array}{@{}rcl@{}} a_{i,n}(x,c)&\lesssim \frac1n\sqrt {cn} e^{-|x-x_{i,n}|\sqrt{cn}/2}, \end{array} $$

2.3 $$ \begin{array}{@{}rcl@{}} \left|\sum\limits_{i=1}^n a_{i}(x,c)-1\right|&\lesssim e^{-x\sqrt {cn}/2}. \end{array} $$

Furthermore, uniformly in *c* and *x* with *c*/*n* ≤ *η*_*n*_ and $\left (x\wedge (1-x)\right ) \sqrt {cn}\ge 2\log n$,

2.4 $$ \begin{array}{@{}rcl@{}} \left|\sum\limits_{i=1}^n a_{i}(x,c)(x-x_{i,n}) \right|&\lesssim \frac 1n. \end{array} $$

Moreover, uniformly in *c* and *x* with *c*/*n* ≤ *η*_*n*_ and $\left (x\wedge (1-x)\right )\sqrt {cn}\ge 1/\eta _{n}$,

2.5 $$ \begin{array}{@{}rcl@{}} \tau_{n}(x,x,c)\sim\frac 14\sqrt{\frac{c}{n}}. \end{array} $$

Also, uniformly in *c* and *x* ≤ *y* with *c*/*n* ≤ *η*_*n*_ and *x* ≥ 1/*n*_+_,

2.6 $$ \begin{array}{@{}rcl@{}} \tau_{n}(x,y,c)&\lesssim&\frac1 {n} \sqrt{cn}\left[1+|x-y|\sqrt{cn}\right] e^{-|x-y|\sqrt{cn}/2}, \end{array} $$

2.7 $$ \begin{array}{@{}rcl@{}} \bar\sigma_{n}(x,y,c) &\lesssim&\frac1{n} \sqrt{cn}\left[1+|x-y|\sqrt{cn}\right] e^{-|x-y|\sqrt{cn}/2}. \end{array} $$

Finally, uniformly in *c* ≤ *d* and *x* ≤ *y* with *d*/*n* ≤ *η*_*n*_ and $\left (x\wedge (1 - x)\right ) \sqrt {cn}\ge 1/\eta _{n}$,

2.8 $$ \begin{array}{@{}rcl@{}} \|\mathbf a_{n}(x,c)-\mathbf a_{n}(y,d)\|^{2}&\lesssim& |x-y|^{2}d \sqrt{dn}+\frac{(c-d)^{2}}{d\sqrt{d n}}, \end{array} $$

2.9 $$ \begin{array}{@{}rcl@{}} {}\bar\sigma_{n}(x,x,c)+\bar\sigma_{n}(y,y,c)-2\bar\sigma_{n}(x,y,c) &\lesssim& |x-y|^{2}c \sqrt{cn}. \end{array} $$

With the help of the preceding lemma we can characterise the order of magnitude of the posterior quantiles used in the construction of the credible bands.

### Proposition 5.

The functions *w*_*n*_ and ${w_{n}^{d}}$ defined by Eqs. 1.6 and 1.8 satisfy $w_{n}(c)\asymp {w_{n}^{d}}(c)\asymp \sqrt {\log (cn)} (c/n)^{1/4}$, uniformly in *c* ∈ *I*_*n*_.

### Proof 1.

The centered posterior process given *c* is Gaussian with covariance function *σ*_*n*_(*x*,*y*,*c*). It can be represented as $\sqrt c W_{x}-\sqrt c \mathbf {W_{n}^{T}}\mathbf a_{n}(x,c)-\mathbf {\varepsilon _{n}^{T}}\mathbf a_{n}(x,c)$. The mean zero Gaussian process $\mathcal {G}$ obtained by replacing the first term $\sqrt c W_{x}$ by $\sqrt c W_{x} 1^{T}\mathbf a_{n}(x,c)$ has covariance function $\bar \sigma _{n}$. By Eq. 2.3 it differs in uniform norm on *x* ∈ *J*_*n*_ no more than of the order $\sup _{x\in J_{n}} \sqrt c e^{-x\sqrt {cn}/2}$$\le \sqrt {c} e^{-\sqrt {cn}/(2l_{n})}$ from the posterior process. This is bounded above by (*c*/*n*)^1/4^ if $(cn)^{1/2}e^{-\sqrt {cn}/l_{n}}\le 1$, in which case the uniform distance between $\mathcal {G}$ and the posterior process would tend to zero faster than (*c*/*n*)^1/4^ and it would suffice to prove that the quantiles of the uniform norm of the process $\mathcal {G}$ behave as claimed. Now the function $s\mapsto s e^{-s/l_{n}}$ is decreasing for *s* ≥ *l*_*n*_. For *c* ∈ *I*_*n*_ we have $s_{n}:=\sqrt {cn}\ge \sqrt {\log n}\gg l_{n}\text {loglog} n$, by the assumption on *l*_*n*_ and hence $(cn)^{1/2}e^{-\sqrt {cn}/l_{n}}\le l_{n}\text {loglog} n e^{-l_{n}\text {loglog} n/l_{n}}=l_{n}\text {loglog} n/\log n\le 1$, uniformly in *c* ∈ *I*_*n*_. We conclude that indeed we may consider $\mathcal {G}$ instead of the posterior process.

By Eq. 2.7 the posterior variance $\bar \sigma _{n}(x,x,c)$ of $\mathcal {G}$ is of the order $\sqrt {c/n}$, uniformly in *x* ∈ *J*_*n*_, while the posterior covariance $\bar \sigma _{n}(x,y,c)$ is much smaller if |*x* − *y*|≫ (*cn*)^− 1/2^.

For a lower bound on the two quantiles *w*_*n*_(*c*) and ${w_{n}^{d}}(c)$, we select a subset *t*_1,*m*_ < ⋯ < *t*_*m*,*m*_ of points from *J*_*n*_ with $t_{i+1,m}-t_{i,m}\ge K(cn)^{-1/2}$, for every *i*, for a sufficiently large constant *K* so that $\bar \sigma _{n}(t_{i,m},t_{j,m},c)\le \delta \bar \sigma _{n}(t,t,c)$, for every *i*,*j* and *t* and some small *δ*. In the case of Eq. 1.8 we choose the points *t*_*i*,*m*_ from the grid points *x*_*i*,*n*_. By Eq. 2.7 we can choose *m* of the order (*cn*)^1/2^. We can lower bound the quantiles of $\max \limits _{i}|\mathcal {G}(t_{i,m})|$ by the expression in the proposition with the help of Lemma 6 below. The quantiles of the supremum of the process $\mathcal {G}$ over its continuous argument are not smaller and hence lower bounded in the same way.

For an upper bound on the quantiles it suffices to show that the mean of the variable $\|\mathcal {G}\|_{\infty }$ is of the order $\sqrt {\log (cn)} (c/n)^{1/4}$. For *x* ∈ *J*_*n*_ and *c* ∈ *I*_*n*_, we have $x\sqrt {cn}\ge l_{n}^{-1}\sqrt {\log n}\rightarrow \infty $, by assumption; similarly $(1-x)\sqrt {cn}\rightarrow \infty $. Therefore by Eq. 2.9 the square of the intrinsic metric of $\mathcal {G}$ satisfies,

$$ d^{2}(x,y)=\bar\sigma_{n}(x,x,c)+\bar\sigma_{n}(y,y,c)-2 \bar\sigma_{n}(x,y,c)\lesssim |x-y|^{2}c\sqrt{cn}. $$

It follows that the diameter for *d* of an interval of Euclidean length proportional to (*cn*)^− 1/2^ is of the order (*c*/*n*)^1/4^ and the *ε*-covering number is bounded above by (*c*/*n*)^1/4^/*ε*. Therefore, for $\|\cdot \|_{\psi _{2}}$ the Orlicz norm relative to $\psi _{2}(x)=e^{x^{2}}-1$, by Corollary 2.2.5 from van der Vaart and Wellner (1996), for any *t*,

$$ \left\|\sup_{x: |x-t|<(cn)^{-1/2}}|\mathcal{G}(x) - \mathcal{G}(t)|\right\|_{\psi_{2}} \!\lesssim\! {\int}_{0}^{(c/n)^{1/4}}\sqrt{\log \left( (c/n)^{1/4}/\varepsilon\right)} d\varepsilon\lesssim (c/n)^{1/4}. $$

Fix a grid (*t*_*i*_) with mesh width (*nc*)^− 1/2^ over *J*_*n*_. Then by the triangle inequality

$$ \mathord\mathrm{E} \sup_{x\in J_{n}}|\mathcal{G}(x)|\lesssim \mathord\mathrm{E} \max_{i}|\mathcal{G}(t_{i})|+\mathord\mathrm{E} \max_{i}\sup_{|x-t_{i}|<(cn)^{-1/2}}|\mathcal{G}(x)-\mathcal{G}(t_{i})|. $$

Here the Orlicz norm of the Gaussian variable $\mathcal {G}(t_{i})$ is bounded by a multiple of its standard deviation $\bar \sigma _{n}(t_{i},t_{i},c)^{1/2}\lesssim (c/n)^{1/4}$. Since there are (*cn*)^1/2^ points *t*_*i*_ in the grid, the preceding display is bounded by $\sqrt {\log (cn)} (c/n)^{1/4}$ by two applications of Lemma 2.2.2 from van der Vaart and Wellner (1996). □

### Lemma 6.

If (*Z*_1,*m*_,…,*Z*_*m*,*m*_) possesses a zero-mean multivariate-normal distribution with ${a_{m}^{2}}\le \mathord \mathrm {E} Z_{i,m}^{2}\lesssim {a_{m}^{2}}$ and $\text {cov}(Z_{i,m}, Z_{j,m})\le \delta {a_{m}^{2}}$ uniformly in (*i*,*j*) for some $a_{m}\rightarrow 0$ and *δ* < 1, then $\Pr \left (\max \limits _{1\le i\le m} |Z_{i,m}|\le w_{m}\right )=\eta \in (0,1)$ implies that $w_{m}\asymp a_{m}\sqrt {\log m}$, as $m\rightarrow \infty $.

### Proof 2.

By Sudakov’s inequality $\mathord \mathrm {E} \max \limits _{i} |Z_{i,m}|\gtrsim a_{m}\sqrt {\log N(c a_{m})}$, for *N*(*ε*) the number of balls of radius *ε* needed to cover the set {1,2,…,*m*} relative to the metric with square *d*^2^(*i*,*j*) = var(*Z*_*i*,*m*_ − *Z*_*j*,*m*_) and any constant *c*. From the assumptions this metric can be seen to satisfy $d^{2}(i,j)\ge 2 {a_{m}^{2}}(1-\delta )$, whence any ball of radius *ca*_*m*_, for *c*^2^ = (1 − *δ*)/2, contains at most one element. Thus *N*(*ca*_*m*_) ≥ *m* and $\mathord \mathrm {E} \max \limits _{i} |Z_{i,m}|\gtrsim a_{m}\sqrt {\log m}$. By the subGaussian maximal inequality this inequality can also be reversed. Furthermore, by Borell’s inequality, for *x* > 0,

$$ \Pr\left( \left|\max_{1\le i\le m} |Z_{i,m}|-\mathord\mathrm{E}\max_{1\le i\le m} |Z_{i,m}|\right|\ge x a_{m}\right) \le 2 e^{-x^{2}/2}. $$

Consequently, for a sufficiently large *x* the distribution of $\max \limits _{i} |Z_{i,m}|$ gives mass arbitrarily close to 1 to an interval of width *a*_*m*_ located in the range $a_{m}\sqrt {\log m}$. Then its nontrivial quantiles must also be in this range. □

## Empirical Bayes estimators

The main result of this section is Proposition 9, which shows that the empirical Bayes estimators $\hat c_{n}$ are contained in an interval around some deterministic value $\tilde c_{n}(f)$, with probability tending to one. This assertion is a slight strengthening of results obtained in Sniekers and van der Vaart (2015a). Its statement and proof require to link the Fourier expansion of a function *f* on the continuous domain [0,1] to its discrete expansion on the design points.

When evaluated at the design points *x*_*i*,*n*_, the eigenbasis *e*_*j*_ in Eq. 1.12 gives rise to an orthogonal basis of $\mathbb {R}^{n}$, which after rescaling to unit length takes the form

3.1 $$  e_{j,n}=\frac{1}{\sqrt{n+1/2}} \left( e_{j}(x_{1,n}),\ldots, e_{j}(x_{n,n})\right)^{T}, \qquad j=1,\ldots, n. $$

The discretisation $\mathbf f_{n}=\left (f(x_{1,n}),\ldots , f(x_{n,n})\right )$ of a function *f* at the design points can be uniquely represented in terms of this basis as $\mathbf f_{n}={\sum }_{j=1}^n f_{j,n}e_{j,n}$, for the coefficients $f_{j,n}:= \mathbf {f_{n}^{T}} e_{j,n}$. If the Fourier series $f(x)={\sum }_{j=1}^{\infty } f_{j}e_{j}(x)$ of the continuous function *f* in terms of the *e*_*j*_ converges pointwise, then the discrete coefficients can be expressed in terms of the coefficients *f*_*j*_ as

3.2 $$ f_{j,n}= \sqrt{n_{+}} \sum\limits_{l=0}^{\infty}(f_{(2n+1)l+j}-f_{(2n+1)l +2n+2-j}). $$

This formula arises through aliasing of higher frequencies: when discretised to the grid of design points each of the continuous basis functions *e*_*j*_ for *j* > *n* coincides with plus or minus $\sqrt {n_{+}}$ times one of the vectors Eq. 3.1 obtained from frequencies *j* ≤ *n*; see Section 4.1 in Sniekers and van der Vaart (2015a) for details. In the latter paper it is shown that the behaviour of the empirical Bayes estimators $\hat {c}_{n}$ given in Eqs. 1.9 and 1.10 is determined by the coefficients (*f*_*j*,*n*_).

Both estimators $\hat c_{n}$ minimise a criterium of the form

$$ c\mapsto D_{1,n}(c,f) + D_{2,n}(c) + R_{n}(c,f), $$

where *D*_1,*n*_ and *D*_2,*n*_ are deterministic, and *R*_*n*_ is a stochastic remainder. With a superscript *L* referring to the likelihood-based and a superscript *R* for the risk-based functions, the deterministic functions are given by

$$ \begin{array}{@{}rcl@{}} D_{1,n}^{L}(c,f)&=& {\sum}_{j=1}^{n}\frac{f_{j,n}^{2}}{(1+c\lambda_{j,n})},\quad D_{2,n}^{L}(c) = {\sum}_{j=1}^n\left[\log(1+c\lambda_{j,n})\!-\frac{c\l_{j,n}}{1+c\lambda_{j,n}}\right],\\ D_{1,n}^{R}(c,f)&=&\sum\limits_{j=1}^{n}\frac{f_{j,n}^{2}}{(1+c\lambda_{j,n})^{2}},\quad D_{2,n}^{R}(c) =\sum\limits_{j=1}^{n}\frac{(c\lambda_{j,n})^{2}}{(1+c\lambda_{j,n})^{2}}, \end{array} $$

where the *λ*_*j*,*n*_ are the eigenvalues of the covariance matrix *U*_*n*_ of standard Brownian motion at the design points; these satisfy *λ*_*j*,*n*_ ≍ *n*/*j*^2^ (Sniekers and van der Vaart (2015a), Example 5). In Sniekers and van der Vaart (2015a) it was proved that for both methods the estimator $\hat {c}_{n}$ minimizes the deterministic part *D*_*n*,1_ + *D*_*n*,2_ within a multiplicative constant that can be chosen arbitrarily close to 1. This is true for general true regression functions *f*. Here we need a more precise result under the assumption that the true function *f* satisfies the *discrete polished tail condition*. For the Brownian motion prior this is the assumption that there exist constants *L* and *ρ* such that, for all sufficiently large *m*,

3.3 $$ \sum\limits_{j=m}^{n} f_{j,n}^{2}\le L\sum\limits_{j=m}^{\rho m\wedge n} f_{j,n}^{2}. $$

### Lemma 7.

If *f* is self-similar of order $\beta >\frac 12$ with constants (*M*,*ε*_1_,*ρ*_1_), then *f* satisfies the discrete polished tail condition Eq. 3.3 with constants (*L*,*ρ*), where *L* and *ρ* depend on (*ε*_1_,*ρ*_1_) only. Moreover, uniformly for *c* ∈ *I*_*n*_ and for proportionality constants that depend on (*ε*_1_,*ρ*_1_) only,

$$ \begin{array}{@{}rcl@{}} D_{1,n}^{L}(c,f) &\asymp M^{2} n \left( \frac{1}{cn}\right)^{\beta},\qquad \text{ if } \beta<1,\\ D_{1,n}^{R}(c,f) &\asymp M^{2} n \left( \frac{1}{cn}\right)^{\beta},\qquad \text{ if } \beta<2. \end{array} $$

The lemma is essentially contained in Sniekers and van der Vaart (2015a); Section 10 of the supplement provides a full proof.

Functions that satisfy the discrete polished tail condition are nicely behaved in the sense that they satisfy the “good bias condition”. The following is Lemma 13 in Sniekers and van der Vaart (2015a).

### Lemma 8 (Good bias condition).

If *f* satisfies the discrete polished tail condition with constants (*L*,*ρ*), then *f* satisfies the *good bias condition* relative to both $D_{1,n}^{L}$ and $D_{1,n}^{R}$: there exists a constant *a* > 0 such that for *c* ∈ *I*_*n*_,

3.4 $$  D_{1,n}(Kc,f)\le K^{-a} D_{1,n}(c,f),\qquad \text{ for all }K>1. $$

The constant *a* can be taken equal to *a* = 2*ρ*(1 + *ρ*)^− 1^(1 + 4*L*)^− 1^.

The functions *D*_2,*n*_ do not depend on *f*, are increasing and behave asymptotically like $\sqrt {cn}$, uniformly in *c* ∈ *I*_*n*_ (Sniekers and van der Vaart (2015a), Lemma 14). The functions *D*_1,*n*_ are clearly decreasing. Let $\tilde {c}_{n}(f)$ be the unique solution to the equation

3.5 $$  D_{1,n}(c,f)=D_{2,n}(c). $$

We have the following result on the location of the empirical Bayes estimators $\hat {c}_{n}$, when the true regression function satisfies the good bias condition and hence in particular when *f* is self-similar.

### Proposition 9.

If *f* satisfies the good bias condition and $\tilde {c}_{n}(f)\in I_n$, then there are positive constants *k* < *K* such that for $\hat c_{n}$ given in Eqs. 1.9 or 1.10

$$ \Pr_{f}\left( \hat{c}_{n}\in[k\tilde{c}_{n}(f),K\tilde{c}_{n}(f)]\right) \rightarrow 1. $$

The constant *k* depends on the constant *a* in the good bias condition only and is increasing in *a*, while *K* is universal. In particular, this is true if *f* is self-similar of order *β* > 1/2; in this case $\tilde c_{n}(f)\asymp M^{4/(1+2\beta )}n^{(1-2\beta )/(1+2\beta )}$, where the proportionality constant depends on the constants (*L*,*ρ*) of self-similarity only.

### Proof 3.

Set *D*_*n*_(*c*,*f*) = *D*_1,*n*_(*c*,*f*) + *D*_2,*n*_(*c*), and for given *ε* > 0 let Λ_*n*_(*ε*) be the set of *c* such that $D_{n}(c,f)\le (1+\varepsilon )\inf _{c\in I_n}D_{n}(c,f)$. By Theorem 12 of Sniekers and van der Vaart (2015b) $\Pr _{f}\left (\hat {c}_{n}\in {\Lambda }_{n}(\varepsilon )\right ) \rightarrow 1$, for every *ε* > 0. Since $\tilde {c}_{n}(f)\in I_n$ by assumption, it follows that $D_{n}(\hat {c}_{n},f)\leq (1+\varepsilon )D_{n}\left (\tilde {c}_{n}(f),f\right )$ with probability tending to one. For the Brownian motion prior it holds that $D_{2,n}(c) \asymp \sqrt {cn}$ (Sniekers and van der Vaart (2015a), Lemma 14). For *f* satisfying the good bias condition, the displayed result then follows by Lemma 17 in Section 11 in the supplement.

If *f* is self-similar of order *β*, then it satisfies the good bias condition by Lemmas 7 and 8. Furthermore, *D*_1,*n*_(*c*,*f*) ≍ *M*^2^*n*(*cn*)^−*β*^, by the first lemma. By monotonicity of the functions *D*_1,*n*_ and *D*_2,*n*_ it then follows that $\tilde c_{n}(f)$ is asymptotically of the same order as the solution in *c* of $M^{2} n (cn)^{-\beta }=\sqrt {cn}$, which is of the order *M*^4/(1 + 2*β*)^*n*^(1 − 2*β*)/(1 + 2*β*)^. □

## Proof of Theorem 1: empirical Bayes case

Denote the centered posterior mean and its bias by

4.1 $$ \begin{array}{@{}rcl@{}} T_{n}(x,c)&=&\hat f_{n,c}(x) - \mathord\mathrm{E}_{f}\hat f_{n,c}(x), \end{array} $$

4.2 $$ \begin{array}{@{}rcl@{}} \mu_{n}(x,c)&=& \mathord\mathrm{E}_{f}\hat f_{n,c}(x) - f(x). \end{array} $$

The proof of Theorem 1 is based on the following two results.

### Lemma 10.

Let *𝜖* > 0. If *f* is Hölder of order *α* ∈ (0,2] with constant *M*, then uniformly in $c\in [n^{-1+\epsilon }, n/\log n]$ and *x* ∈ *J*_*n*_,

$$ \left|\mu_{n}(x,c)\right| \lesssim M\left( \frac{1}{cn}\right)^{\alpha/2}\vee \frac Mn. $$

### Proof 4.

By the definition of the coefficients *a*_*i*,*n*_ we have $\mu _{n}(x,c) = {\sum }_{i=1}^{n} a_{i,n}(x,c)f(x_{i,n}) -f(x)$. By Eq. 2.3 in Lemma 4 we have that ${\sum }_{i=1}^{n} a_{i,n}$$(x,c) =1+o\left ((cn)^{-\alpha /2}\right )$ for $x\gg \alpha (cn)^{-1/2}\log (cn)$, which is valid if *x* ∈ *J*_*n*_ and *c* ∈ *I*_*n*_. Therefore it suffices to bound the function $ \tilde \mu _{n}(x,c) = {\sum }_{i=1}^{n} a_{i,n}(x,c)\left (f(x_{i,n}) -f(x)\right )$.

For *α* ∈ (0,1] and *f* with *α*-Hölder norm bounded by *M* the absolute value of $\tilde \mu _{n}(x,c)$ is bounded above by

$$ \begin{array}{@{}rcl@{}} &&\sum\limits_{i=1}^{n} a_{i,n}(x,c) |f(x_{i,n})-f(x)|\leq M \sum\limits_{i=1}^{n} a_{i,n}(x,c) |x_{i,n}-x|^{\alpha}\\ &&\qquad\lesssim\int\sqrt{cn} e^{-|s-x|\sqrt{cn}/2}|s-x|^{\alpha} ds+\frac 1n \sup_{s}\sqrt{cn} e^{-|s-x|\sqrt{cn}/2}|s-x|^{\alpha}, \end{array} $$

by Eq. 2.2 and Lemma 18, uniformly in *c* ∈ *I*_*n*_ and *x* ∈ *J*_*n*_. This is of the order (*cn*)^−*α*/2^ and proves the result.

If *f* is *α*-Hölder for *α* = 1 + *δ* and some *δ* ∈ (0,1], then by the mean value theorem there exist *ξ*_*i*,*n*_ between *x*_*i*,*n*_ and *x* so that $f(x_{i,n})-f(x)=f^{\prime }(\xi _{i,n})(x_{i,n}-x)$, and hence

$$ \begin{array}{@{}rcl@{}} |\tilde \mu_{n}(x,c)| &\le& \left|\sum\limits_{i=1}^{n} a_{i,n}(x,c) \left( f^{\prime}(\xi_{i,n})-f^{\prime}(x)\right) (x_{i,n}-x)+ f^{\prime}(x)\sum\limits_{i=1}^{n} a_{i,n}(x,c)(x_{i,n}-x)\right|\\ &\leq& M \sum\limits_{i=1}^{n} a_{i,n}(x,c) |x_{i,n}-x|^{1+\delta} + M\left|\sum\limits_{i=1}^{n} a_{i,n}(x,c)(x_{i,n}-x)\right|. \end{array} $$

By the argument in the preceding paragraph the first term can be seen to be bounded by a multiple of (*cn*)^−(1+*δ*)/2^. The second term is bounded by 1/*n*, by Eq. 2.4 of Lemma 4, for any *c* ≥ *n*^− 1+*ε*^ and *x* ∈ *J*_*n*_. □

### Proposition 11.

If *f* satisfies the good bias condition and $\tilde {c}_{n}(f)\in I_n$, then for both the risk-based and likelihood-based empirical Bayes estimators $\hat {c}_{n}$,

$$ \sup_{x\in J_n}\frac{|T_{n}(x,\hat{c}_{n})|}{\sqrt{\log (\hat c_{n}n)}(\hat{c}_{n}/n)^{1/4}} =O_{P_{f}}(1). $$

This statement is uniform in *f* such that the constant *a* in the good bias condition is bounded.

### Proof 5.

Since *f* satisfies the good bias condition and $\tilde {c}_{n}:=\tilde c_{n}(f)$ is contained in *I*_*n*_, it follows by Proposition 9 that $\Pr _{f}\left (\hat {c}_{n}\in [k\tilde {c}_{n},K\tilde {c}_{n}]\right ) \rightarrow 1$, for some positive constants *k* < *K*. The constant *k* depends on the constant *a* in the good bias condition only and is bounded in *a*, while *K* is universal. Thus it suffices to show that the variables $\sup _{x\in J_n}\sup _{c\in [k\tilde {c}_n,K\tilde {c}_n]}|T_{n}(x,c)|$ are of the order $\sqrt {\log (\tilde c_{n}n)}(\tilde c_{n}/n)^{1/4}$ in probability.

Denote by $\|\cdot \|_{\psi _{2}}$ the Orlicz norm corresponding to the function $\psi _{2}(x)=e^{x^{2}}-1$. Let *t*_1,*n*_ < *t*_2,*n*_ < ⋅ < *t*_*m*,*n*_ be a minimal set of points over *J*_*n*_ that includes the two endpoints and hence meshwidth bounded by $1/\sqrt {\tilde c_{n} n}$; hence $m\sim \sqrt {\tilde c_{n} n}$. By Lemma 2.2.2 from van der Vaart and Wellner (1996),

$$ \begin{array}{@{}rcl@{}} \mathord\mathrm{E} \sup_{x\in J_n}\sup_{c\in[k\tilde{c}_n,K\tilde{c}_n]}|T_{n}(x,c)| &&\lesssim \sqrt{\log m} \max_{i\in\{1,\dots,m-1\}} \\&&\left\| \sup_{x\in(t_{i,n},t_{i+1,n}]}\sup_{c\in[k\tilde{c}_n,K\tilde{c}_n]}|T_{n}(x,c)| \right\|_{\psi_{2}}. \end{array} $$

It suffices to show that the norms on the right are of the order $(\tilde c_{n}/n)^{1/4}$. The stochastic process *T*_*n*_ is zero-mean Gaussian with

$$ \begin{array}{@{}rcl@{}} \text{var}\left[T_{n}(x_{1},c_{1})-T_{n}(x_{2},c_{2})\right] &= \| \mathbf{a}_{n}(x_{1},c_{1})-\mathbf{a}_{n}(x_{2},c_{2}) \|^{2} \lesssim \frac{(c_{1}-c_{2})^{2}}{\tilde{c}_{n}^{3/2}\sqrt n} + \sqrt n (x_{1}-x_{2})^{2} \tilde{c}_{n}^{3/2}, \end{array} $$

by Eq. 2.8 of Lemma 4, for every *x*_1_ < *x*_2_ ∈ (*t*_*i*,*n*_,*t*_*i*+ 1,*n*_] and $c_{1}<c_{2}\in [k\tilde {c}_n,K\tilde {c}_n]$. Let $d\left ((x_{1},c_{1}),(x_{2},c_{2})\right )$ be the root of the right side. Since ${\tilde {c}_{n}}/{n}\rightarrow 0$, the diameter of the set $T:=(t_{i,n},t_{i+1,n}]\times [k\tilde {c}_n,K\tilde {c}_n]$ for *d* is bounded above by a multiple of $(\tilde c_{n}/n)^{1/4}$, and the *ε*-covering number of *T* is bounded by

$$ N(\varepsilon,d) \lesssim \frac{1/\sqrt{\tilde c_{n}n}}{\varepsilon/(\tilde{c}_{n}^{3/4}n^{1/4})}\frac{\tilde{c}_{n}}{\varepsilon\tilde{c}_{n}^{3/4}n^{1/4}} = \frac{\sqrt{\tilde{c}_{n}/n}}{\varepsilon^{2}}. $$

Applying Corollary 2.2.5 in van der Vaart and Wellner (1996), we obtain, for every *i*,

$$ \left\| \sup_{(x_{1},c_{1}),(x_{2},c_{2})\in T}|T_{n}(x_{1},c_{1})-T_{n}(x_{2},c_{2})| \right\|_{\psi_{2}} \lesssim {\int}_{0}^{(\tilde{c}_{n}/n)^{1/4}}\sqrt{\log\left( 1+\frac{\sqrt{\tilde{c}_{n}/n}}{\varepsilon^{2}}\right)} \text{d}\varepsilon \asymp (\tilde c_{n}/n)^{1/4}. $$

Combining this with the fact that $\| T_{n}(x_{0},c_{0})\|_{\psi _{2}}\!\lesssim \! \sqrt {\text {var} T_{n}(x_{0},c_{0})} \!\lesssim \! (\tilde {c}_{n}/n)^{1/4}$, for any fixed (*x*_0_,*c*_0_) in *T*, by Eq. 2.5, we see that $\left \| \sup _{(x,c)\in T}|T_{n}(x,c)| \right \|_{\psi _{2}}\lesssim (\tilde {c}_{n}/n)^{1/4}$, and hence it has the desired order of magnitude. □

We are ready for the proof of Theorem 1 and the first assertion of Theorem 3.

In view of Proposition 5, the function *f* is contained in $\hat C_{n}(L)$ for a sufficiently large constant *L* if and only if we have $\sup _{x\in J_n} |f(x)- \hat f_{n,\hat {c}_{n}}(x)| < L\sqrt {\log (\hat c_{n} n)}\left (\hat {c}_{n}/n\right )^{1/4}$. By the triangle inequality this is certainly the case if

4.3 $$ \frac{\sup_{x\in J_n}|T_{n}(x,\hat{c}_{n})| + \sup_{x\in J_n} |\mu_{n}(x,\hat{c}_{n})|}{L\sqrt{\log (\hat c_{n}n)}\left( \hat{c}_{n}/n\right)^{1/4}} < 1. $$

If *f* is self-similar of order $\beta >\frac 12$, then the array (*f*_*i*,*n*_) is discrete polished tail by Lemma 7, and hence *f* satisfies the good bias condition by Lemma 8. Moreover, by Proposition 9 we have $\tilde {c}_{n}(f) \asymp M^{4/(1+2\beta )}n^{({1-2\beta })/({1+2\beta })}$. Since $\tilde {c}_{n}\in I_n$, it follows by Proposition 11 that the first term of Eq. 4.3 can be made arbitrarily small by choice of *L*. Moreover, by Proposition 9 there are positive constants *k* < *K* such that $\hat {c}_{n}\in [k\tilde {c}_{n}(f),K\tilde {c}_{n}(f)]$ with probability tending to one. Consider the second term in Eq. 4.3. Since *f* ∈ *C*^*α*^[0,1], we have

$$ |\mu_{n}(x,c)|^{2} \lesssim M^{2}\left( \frac{1}{cn}\right)^{\alpha} $$

uniformly for $c\in [k\tilde {c}_{n}, K \tilde {c}_{n}]$ and *x* ∈ *J*_*n*_ by Lemma 10. We see that for *α* ≥ *β* we have

$$ \sup_{x\in J_n}|\mu_{n}(x,\hat{c}_{n})|^{2} \lesssim M^{2}\left( \frac{1}{\hat{c}_{n} n}\right)^{\alpha} \lesssim M^{2}\left( \frac{1}{\hat{c}_{n} n}\right)^{\beta} M^{2}\asymp \left( \frac{1}{\tilde{c}_{n}(f) n}\right)^{\beta} \lesssim \sqrt{\frac{\tilde{c}_{n}(f)}{n}} \asymp \sqrt{\frac{\hat{c}_{n}}{n}} $$

with probability tending to one. It follows that the second term in Eq. 4.3 tends to 0 with probability tending to one. This concludes the proof of Theorem 1 for the empirical Bayes intervals.

Since $(\tilde {c}_{n}(f)/n)^{1/4}\asymp n^{-\beta /(1+2\beta )}$, the diameter of the credible band is of the order $\sqrt {\log n} n^{-\beta /(1+2\beta )}$ with probability tending to one, in view of Proposition 5. This proves the first assertion of Theorem 3.

## Proof of Theorem 2: empirical Bayes case

We shall obtain Theorem 2 as a corollary of the following theorem, which guarantees coverage for a deterministic set of functions that will be shown to contain the random functions in the theorem with probability one.

For given *L*_0_ > 0 let $\mathcal {F}_{L_{0},a}$ be the set of all functions *f* that satisfy the good bias condition Eq. 3.4 with constant *a* and for which there exists $N\in \mathbb {N}$ such that for every *n* ≥ *N* the point $\tilde c_{n}(f)$ that equalises *D*_1,*n*_(*c*,*f*) and *D*_2,*n*_(*c*) (see Eq. 3.5) is contained in *I*_*n*_, and such that

5.1 $$ \max_{j\in\mathcal{J}_{n}} \frac{1}{n_{+}}\left( \sum\limits_{i=1}^{n} \frac{f_{i,n} e_{i}(x_{j,n})}{1+\tilde{c}_{n}(f)\lambda_{i,n}}\right)^{2} < L_{0} \log (\tilde c_{n}(f)n)\sqrt{\frac{\tilde{c}_{n}(f)}{n}}. $$

In the proof below the expression on the left side is shown to be the maximum of the square bias at the design points. Thus the condition simply assumes that the bias is smaller than the posterior deviation (a property that we proved in the preceding section under a Hölder condition on *f*, but will be seen to hold also for functions generated from the prior).

We shall describe the width of the credible band using the norms, for *β* > 0,

$$ \begin{array}{@{}rcl@{}} \|f\|_{n,\beta}^{2}=\frac 1n{\sum}_{j=1}^n j^{2\beta}f_{j,n}^{2}, \qquad \|f\|_{n,\beta,\infty}^{2}=\frac 1n\sup\limits_{1\le j\le n}j^{1+2\beta}f_{j,n}^{2}. \end{array} $$

### Theorem 12.

For every *L*_0_ > 0 and for both the risk-based and likelihood-based empirical Bayes methods there exists *L* such that the credible sets ${C_{n}^{d}}(\hat c_{n}, L)$ as in Eq. 1.7 satisfy

$$ \liminf_{n\rightarrow\infty} \inf_{f\in\mathcal{F}_{L_{0},a}} \Pr_{f}\left( f\in {C_{n}^{d}}(\hat c_{n},L)\right)\ge \eta. $$

Furthermore, for given *β* < 1 the diameter of the credible set ${C_{n}^{d}}(\hat c_{n},L)$ is of the order $O_{P}\left (\sqrt {\log n} n^{-\beta /(1+2\beta )}\right )$ uniformly in *f* with $\|f\|_{n,\beta }\lesssim 1$ or $\|f\|_{n,\beta ,\infty }\lesssim 1$. For the risk-based empirical Bayes method this is also true for *β* < 2.

### Proof 6.

As before let *T*_*n*_(*x*,*c*) be the posterior mean $\hat f_{n,c}(x)$ minus its expectation and let *μ*_*n*_(*x*,*c*) the bias of the posterior mean (see Eq. 4.1– Eq. 4.2). The function *f* is covered by $\hat {C_{n}^{d}}(\hat c_{n},L)$ for a sufficiently large *L* if we have

5.2 $$ \frac{\max_{i \in \mathcal{J}_{n}}|T_{n}(x_{i,n},\hat{c}_{n})| + \max_{i \in \mathcal{J}_{n}} |\mu_{n}(x_{i,n},\hat{c}_{n})|} {L\sqrt{\log (\hat c_{n}n)}\left( \hat{c}_{n}/n\right)^{1/4}} < 1. $$

The term involving $T_{n}(x_{i,n},\hat c_{n})$ can be bounded by *L*^− 1^ times the supremum in Proposition 11, and hence gives a contribution that is arbitrarily small if *L* is sufficiently large. We need to control the second term involving the bias at the grid points.

The vector **f**_*n*_ has prior *N*(0,*cU*_*n*_). Therefore its coordinates *f*_*i*,*n*_ relative to the eigenbasis *e*_*j*,*n*_ of *U*_*n*_ are a priori independent, and have *N*(0,*c*ł_*i*,*n*_)-priors. The coordinates $\tilde Y_{i,n}$ of the observation **Y**_*n*_ relative to the same basis are independent *N*(*f*_*i*,*n*_,1) variables. It follows that under the posterior distribution the coordinates *f*_*i*,*n*_ are again independent, and have normal distributions with means $c\l _{i,n}/(1+c\l _{i,n})\tilde Y_{i,n}$. Hence the expectation of the posterior mean of *f*_*i*,*n*_ is equal to *c*ł_*i*,*n*_/(1 + *c*ł_*i*,*n*_)*f*_*i*,*n*_.

For a grid point *x*_*j*,*n*_ we have the representation $f(x_{j,n}) = {\sum }_{i=1}^{n} f_{i,n} (e_{i,n})_{j}$$= n_{+}^{-1/2}{\sum }_{i=1}^{n} f_{i,n}e_{i}(x_{j,n})$. The posterior mean of *f*(*x*_*j*,*n*_) is obtained by replacing the *f*_*i*,*n*_ by their posterior means. In combination with the preceding paragraph this shows that the bias at the grid point *x*_*j*,*n*_ can be written in terms of the coefficients (*f*_*i*,*n*_) as

$$ \begin{array}{@{}rcl@{}} \mu_{n}(x_{j,n},c) =\mathord\mathrm{E}_{f}\sum\limits_{i=1}^{n} \left[\frac {c\l_{i,n}}{1+c\l_{i,n}}\tilde Y_{i,n}- f_{i,n}\right] (e_{i,n})_{j} = -\frac{1}{\sqrt{n_{+}}} \sum\limits_{i=1}^{n} \frac{f_{i,n} e_{i}(x_{j,n})}{1+c\lambda_{i,n}}. \end{array} $$

Assumption Eq. 5.1 entails that the square of this expression at $c=\tilde c_{n}(f)$ is bounded uniformly in $j\in \mathcal {J}_{n}$ by $L_{1} \log (\tilde c_{n}(f)n)\sqrt {\tilde c_{n}(f)/n}$, for some constant *L*_1_. This is then also true uniformly for $c\in [k\tilde {c}_n,K\tilde {c}_n]$, for any constants *K* > *k* > 0. By Proposition 9 $\hat c_{n}$ falls in this interval with probability tending to one. It follows that the second term in Eq. 5.2 can be made arbitrarily small with probability tending to one, by taking *L* to be sufficiently large.

For functions *f* with $\|f\|_{n,\beta }\lesssim 1$ or $\|f\|_{n,\beta ,\infty }\lesssim 1$ we have that $D_{1,n}^{R}(c,f)\lesssim n(cn)^{-\beta }$ if *β* < 2, while $D_{1,n}^{L}(c,f)\lesssim n(cn)^{-\beta }$ if *β* < 1. (See Examples 19 and 22 in Sniekers and van der Vaart (2015a) or Eqs. 10.3 and 10.4.) Since $D_{2,n}(c)\asymp \sqrt {cn}$, this implies that $\tilde {c}_{n}(f)\lesssim n^{(1-2\beta )/(1+2\beta )}$. Then the assertions on the diameter follow from Proposition 5, since $\hat c_{n}\asymp c_{n}(f)$. □

We can now prove Theorem 2 by showing that *W* is in $\mathcal {F}_{L_{0},a}$ for some *L*_0_,*a*, almost surely. We give the proof for the risk-based empirical Bayes method; the proof for the likelihood-based method is analogous. The following lemma is Proposition 36 in Sniekers and van der Vaart (2015a).

### Lemma 13.

The coordinates *W*_*i*,*n*_ of the restriction **W**_*n*_ of the process Eq. 1.14 to the grid points relative to the basis *e*_*i*,*n*_ are independent normal random variables with zero mean and var(*W*_*i*,*n*_) ≍ *ni*^− 1 − 2*α*^. Moreover, for sufficiently large *L* and *ρ* almost every realisation of *W* is discrete polished tail Eq. 3.3 with constants (*L*,*ρ*).

By Lemma 13 almost every realisation of *W* is discrete polished tail, and hence satisfies the good bias condition, by Lemma 8. We must prove that almost surely there is an *N* and *L*_0_ such that $\tilde {c}_{n}(W)\in I_n$ and such that Eq. 5.1 holds for *n* ≥ *N*.

For the proof of the first consider the stochastic process

$$ V_{n}(c) = \frac{1}{n(cn)^{-\alpha}} \left( D_{1,n}^{R}(c,W) - \mathord\mathrm{E} D_{1,n}^{R}(c,W)\right) = \frac{1}{n(cn)^{-\alpha}} \sum\limits_{i=1}^{n} \frac{W_{i,n}^{2} - \mathord\mathrm{E} W_{i,n}^{2}}{(1+c\lambda_{i,n})^{2}}. $$

In view of Lemma 13,

$$ \begin{array}{@{}rcl@{}} \mathord\mathrm{E} D_{1,n}^{R}(c,W) &\asymp& {\sum}_{i=1}^{n} \frac{ni^{3-2\alpha}}{(i^{2}+cn)^{2}} \asymp n(cn)^{-\alpha},\\ \text{var}\left[V_{n}(c)\right] &\asymp& (cn)^{2\alpha} {\sum}_{i=1}^{n} \frac{i^{6-4\alpha}}{(i^{2}+cn)^{4}} \asymp \left\{\begin{array}{lll} (cn)^{-1/2}, &\text{ if } \alpha<7/4,\\ (cn)^{-1/2}\log(cn), &\text{ if } \alpha=7/4,\\ (cn)^{2\alpha-4},&\text{ if } \alpha>7/4. \end{array}\right. \end{array} $$

Therefore there exist constants 0 < *γ*_1_ < Γ_1_ such that, for *c* ∈ *I*_*n*_,

$$ n(cn)^{-\alpha} \left( \gamma_{1} + V_{n}(c)\right) \leq D_{1,n}^{R}(c,W) \leq n(cn)^{-\alpha} \left( {\Gamma}_{1} + V_{n}(c)\right). $$

Also (see Lemma 14 in Sniekers and van der Vaart (2015a)) there exist constants 0 < *γ*_2_ < Γ_2_ such that

$$ \gamma_{2}\sqrt{cn}\leq D_{2,n}^{R}(c) \leq {\Gamma}_{2}\sqrt{cn}. $$

Set *b* := (1 − 2*α*)/(1 + 2*α*) and consider the event $E_{n} = \left \{\tilde {c}_{n}(W)\notin \left [k n^{b},Kn^{b}\right ]\right \}$. Because *D*_1,*n*_ is nonincreasing and *D*_2,*n*_ is nondecreasing,

$$ \begin{array}{@{}rcl@{}} \Pr\left( \tilde{c}_{n}(W) < k n^{b}\right) &\leq& \Pr\left( D_{1,n}^{R}\left( k n^{b},W\right) < D_{2,n}^{R}\left( k n^{b}\right)\right)\\ &\leq& \Pr\left( k^{-\alpha}n^{1/(1+2\alpha)}\left[\gamma_{1} + V_{n}\left( k n^{b}\right)\right] \!<\! {\Gamma}_{2}\sqrt{k}n^{1/(1+2\alpha)}\right) \\&&=\Pr\left( V_{n}\left( k n^{b}\right) < -a\right), \end{array} $$

where *a* := *γ*_1_ −Γ_2_*k*^1/2+*α*^ is positive for sufficiently small *k*. Denote by $\|\cdot \|_{\psi _{1}}$ the Orlicz norm corresponding to the function *ψ*_1_(*x*) = *e*^*x*^ − 1. Applying Proposition A.1.6 in van der Vaart and Wellner (1996) with $X_{i}= (W_{i,n}^{2} - \mathord \mathrm {E} W_{i,n}^{2})/(n(cn)^{-\alpha }(1+c\lambda _{i,n})^{2})$, *S*_*n*_ = *V*_*n*_(*c*) and *p* = 1, followed by Lemma 2.2.2 from the same book, we see that

$$ \begin{array}{@{}rcl@{}} \|V_{n}(c)\|_{\psi_{1}} & \lesssim& \|V_{n}(c)\|_{1} + \left\| \max_{i \in \mathcal{J}_{n}} |X_{i}| \right\|_{\psi_{1}}\\ &\lesssim& \sqrt{\mathord\mathrm{E} \left[V_{n}(c)\right]^{2}} + \frac{\log n}{n(cn)^{-\alpha}} \max_{i \in \mathcal{J}_{n}}\frac{\|W_{i,n}^{2} - \mathord\mathrm{E} W_{i,n}^{2}\|_{\psi_{1}}}{(1+c\lambda_{i,n})^{2}}. \end{array} $$

Since *W*_*i*,*n*_ is a mean zero normal random variable with variance of the order *ni*^− 1 − 2*α*^, it follows by Lemma 2.2.1 from van der Vaart and Wellner (1996) that $\|W_{i,n}^{2} - \mathord \mathrm {E} W_{i,n}^{2}\|_{\psi _{1}} \leq 2 \|W_{i,n}^{2}\|_{\psi _{1}} \lesssim n i^{-1-2\alpha }$. We conclude that

$$ \begin{array}{@{}rcl@{}} \max_{i \in \mathcal{J}_{n}}\frac{\|W_{i,n}^{2} - \mathord\mathrm{E} W_{i,n}^{2}\|_{\psi_{1}}}{(1+c\lambda_{i,n})^{2}} \lesssim n \max_{i \in {\cal J}_{n}}\frac{i^{3-2\alpha}}{(i^{2}+cn)^{2}} \lesssim \left\{\begin{array}{ll} n(cn)^{-1/2-\alpha} &\!\text{ if } \alpha\leq 3/2,\\ n(cn)^{-2} &\!\text{ if } \alpha>3/2. \end{array}\right. \end{array} $$

Combining the above results, it follows that there is a constant *C* > 0 such that

$$ \begin{array}{@{}rcl@{}} \|V_{n}\left( k n^{b}\right)\|_{\psi_{1}} & \le \left\{\begin{array}{ll} C n^{-\frac14(1+b)}, &\!\text{ if } \alpha<7/4,\\ C n^{-(2-\alpha)(1+b)} \log n,&\!\text{ if } \alpha\ge 7/4. \end{array}\right. \end{array} $$

Therefore, by Markov’s inequality,

$$ \begin{array}{@{}rcl@{}} \Pr\left( V_{n}\left( k n^{b}\right) < -a\right) &\leq \frac{1}{\psi_{1}\left( a/\|V_{n}\left( k n^{b}\right)\|_{\psi_{1}}\right)} \leq \left\{\begin{array}{ll} \left( e^{\frac{a}{C}n^{\frac14(1+b)}}-1\right)^{-1}, &\!\text{ if } \alpha<7/4,\\ \left( e^{\frac{a}{C\log n}n^{(2-\alpha)(1+b)}}-1\right)^{-1}, &\!\text{ if } \alpha\ge 7/4. \end{array}\right. \end{array} $$

We conclude that there are constants $\tilde {K},\tilde {k},\gamma >0$ such that for *α* ∈ (0,2) it holds that $\Pr \left (V_{n}\left (k n^{b}\right ) \!<\!-a\right )\leq \tilde {K} e^{-\tilde {k} n^{\gamma }}$. The probability $ \Pr \left (\tilde {c}_{n}(W) \!>\! K n^{b}\right )$ can be bounded by a similar argument. Consequently,

$$ \begin{array}{@{}rcl@{}} \sum\limits_{n=1}^{\infty} \Pr(E_{n}) = \sum\limits_{n=1}^{\infty}\Pr\left( \tilde{c}_{n}(W) < k n^{b}\right) + \sum\limits_{n=1}^{\infty} \Pr\left( \tilde{c}_{n}(W)>K n^{b}\right) \lesssim \sum\limits_{n=1}^{\infty} e^{-\tilde{k} n^{\gamma}} < \infty. \end{array} $$

Then the Borel-Cantelli lemma gives that almost surely there is an *N* such that $\tilde {c}_{n}(W)\in \left [k n^{b},Kn^{b}\right ]$ and hence $\tilde {c}_{n}(W)\in I_n$ for *n* ≥ *N*.

Finally we prove that condition Eq. 5.1 is satisfied almost surely. The right side of this condition is of the order $\log n\sqrt {\tilde c_{n}(W)/n}\lesssim (\log n)n^{-\alpha /(1+2\alpha )}$. Define

$$ U_{n}(x,c) := \frac{1}{\sqrt{n_{+}}}\sum\limits_{i=1}^{n} \frac{W_{i,n} e_{i}(x)}{1+c\lambda_{i,n}}. $$

By Lemma 13, uniformly for *x* ∈ (0,1), *c* ∈ *I*_*n*_, and *s* < *t* ∈ *I*_*n*_

$$ \begin{array}{@{}rcl@{}} \text{var}\left[ U_{n}(x,c)\right] &\lesssim& \sum\limits_{i=1}^{n} \frac{i^{3-2\alpha}}{(i^{2}+cn)^{2}} \lesssim (cn)^{-\alpha},\\ \text{var}\left[U_{n}(x,s) - U_{n}(x,t)\right] & = \!& \frac{1}{n_{+}}\sum\limits_{i=1}^{n} \!\text{var}(W_{i,n})e_{i}(x)^{2}\left[\frac{1}{1+s\lambda_{i,n}}-\frac{1}{1+t\lambda_{i,n}}\right]^{2}\\ &\lesssim& \frac{(s-t)^{2}}{s^{2}} \sum\limits_{i=1}^{n} \frac{i^{3-2\alpha}}{(i^{2}+sn)^{2}} \lesssim \frac{(s-t)^{2}}{s^{2}} (sn)^{-\alpha}. \end{array} $$

Denote by $\|\cdot \|_{\psi _{2}}$ the Orlicz norm corresponding to the function $\psi _{2}(x)=e^{x^{2}}-1$. Since *U*_*n*_(*x*,*s*) − *U*_*n*_(*x*,*t*) has a normal distribution with mean zero, we see that, for $s<t\in \left [k n^{b},K n^{b}\right ]$ and uniformly for *x* ∈ (0,1),

$$ \left\|U_{n}(x,s)-U_{n}(x,t)\right\|^{2}_{\psi_{2}} \lesssim \text{var}\left[U_{n}(x,s)-U_{n}(x,t)\right] \lesssim n^{-2b} n^{-\alpha(1+b)} (s-t)^{2}. $$

It then follows by Corollary 2.2.5 in van der Vaart and Wellner (1996) with $T=\left [k n^{b},K n^{b}\right ]$ that

$$ \begin{array}{@{}rcl@{}} \left\| \sup_{s,t\in [k n^{b},K n^{b}]}|U_{n}(x,s)-U_{n}(x,t)|\right\|_{\psi_{2}} &\!\!\lesssim& n^{-b-\frac{\alpha}{2}(1+b)}\! {\int}_{0}^{n^{b}}\!\!\sqrt{\log\left( \!1+\frac{n^{b}}{\varepsilon}\!\right)} \text{d}\varepsilon\\ & \!\le& \!n^{-b-\frac{\alpha}{1+2\alpha}}\! {\int}_{0}^{n^{b}}\!\!\sqrt{\frac{n^{b}}{\varepsilon}} \text{d}\varepsilon = n^{-\alpha/(1+2\alpha)}. \end{array} $$

Applying Lemma 2.2.2 from van der Vaart and Wellner (1996) and noting that $\left \| U_{n}(x,c)\right \|_{\psi _{2}}\lesssim \sqrt {\text {var} U_{n}(x,c)} \lesssim n^{-\alpha /(1+2\alpha )}$ for any fixed $c \in \left [k n^{b},K n^{b}\right ]$, we see that there exists a constant $\tilde {C}>0$ with

$$ \begin{array}{@{}rcl@{}} \mathord\mathrm{E} \max_{j \in \mathcal{J}_{n}}\sup_{c\in [k n^{b},K n^{b}]}|U_{n}(x_{j,n},c)| \leq \tilde{C} \sqrt{\log n} \cdot n^{-\frac{\alpha}{1+2\alpha}}. \end{array} $$

By Borell’s inequality the variable $S_{n}=\max \limits _{j \in \mathcal {J}_{n}}\sup _{c\in [k n^{b},K n^{b}]}|U_{n}(x_{j,n},c)|$ satisfies

$$ \Pr\left( \left|S_{n}-\mathord\mathrm{E} S_{n}\right|> t \sqrt{\log n} \tilde C (cn)^{-\alpha/2}\right) \le 2 e^{-t^{2}\log n}. $$

For sufficiently large *t* the right side is summable over *n*, whence the limsup of the events has probability zero. Since $(cn)^{-\alpha /2}\sim n^{-\alpha /(1+2\alpha )}$, for *c* ∈ [*kn*^*b*^,*Kn*^*b*^], combination of the two preceding displays gives that $S_{n}\lesssim \sqrt {\log n} n^{-\alpha /(1+2\alpha )}$ eventually, almost surely. Because $\tilde c_{n}(W)$ is of polynomial order in *n*, the factors $\log n$ and $\log (\tilde c_{n}(W) n)$ are equivalent up to constants. This implies Eq. 5.1, and concludes the proof of Theorem 2 for the empirical Bayes choice of *c*.

By Lemma 13 the square norm $\|W\|_{n,\beta }^{2}$ of the stochastic process *W* has expectation of the order ${\sum }_{j} j^{2\beta -1-2\alpha }$, which is finite for *β* < *α*. Together with the last assertion of Theorem 12 this implies the last assertion of Theorem 3.

## Proof of Theorems 1 and 2: hierarchical Bayes case

In this section we extend the proofs of the main results to the hierarchical Bayes method. The key is the following result from Sniekers and van der Vaart (2015a) (see Theorem 25), which shows that the posterior distribution of the smoothing parameter *c* concentrates near the likelihood-based empirical Bayes estimator. Recall that the prior density for *c* is given by Eq. 1.11.

### Proposition 14.

Suppose that there is a minimiser *c*_*n*_(*f*) of $c\mapsto {D_{n}^{L}}(c,f)+2\lambda /c$ over $c\in (0,\infty )$ that satisfies *c*_*n*_(*f*) ∈ *I*_*n*_ and 2*c*_*n*_(*f*) ∈ *I*_*n*_. Then there exist constants $0<k<K<\infty $ such that

6.1 $$  {\Pi}_{n}\left( c: c\in \left[k c_{n}(f), Kc_{n}(f)\right] | \mathbf Y_{n} \right) \stackrel{P_{f}}{\rightarrow} 1. $$

We have that $f\in \hat C_{n}(L)$ as in Theorem 1 if and only if there exists $c\in (\hat {c}_{1,n},\hat {c}_{2,n})$ such that *f* satisfies

6.2 $$ \sup_{x\in J_n} |f(x)- \hat f_{n,c}(x)|<L\sqrt{\log (cn)}\left( \frac{c}{n}\right)^{1/4}. $$

In Section 4 this display was seen to be valid for every *c* in an interval $\left [k \tilde {c}_{n}(f), K\tilde {c}_{n}(f)\right ]$ around the point $\tilde c_{n}(f)$ that equalises the functions $c\mapsto D_{n,1}^{L}(c,f)$ and $c\mapsto D_{n,2}^{L}(c)$. Thus it suffices to show that an interval of this form contains a point from $(\hat {c}_{1,n},\hat {c}_{2,n})$. Since the interval $(\hat {c}_{1,n},\hat {c}_{2,n})$ has positive posterior mass by construction, it certainly suffices to show that Eq. 6.1 is valid with *c*_*n*_(*f*) replaced by $\tilde c_{n}(f)$.

Now for every $f\in \mathcal {F}_{\alpha ,\beta }$ Lemma 7 gives that

$$ D_{n,1}^{L}(c,f)+ D_{n,2}^{L}(c)+\frac{2\lambda}{c}\asymp n\left( \frac{1}{cn}\right)^{\beta}+ \sqrt{cn}+ \frac{2\lambda}{c}. $$

By definition $\tilde {c}_{n}(f)$ equalises the first two terms. From the explicit expression on the right side it is seen that the minimiser of the sum of the first two terms is of the same order as the equaliser and both are of the order *n*^(1 − 2*β*)/(1 + 2*β*)^. Since the last term is of smaller order in $\tilde {c}_{n}(f)$, we conclude that the minimiser *c*_*n*_(*f*) of the entire expression is of the same order as well. Thus $c_{n}(f)\asymp \tilde c_{n}(f)$ and hence the desired result follows by Proposition 14.

For the proof in the case of Theorem 2 we apply the same argument, but now show that Eq. 6.1 is valid with *c*_*n*_(*f*) replaced by $\tilde {c}_{n}(W)$, almost surely. This is true as both $\tilde c_{n}(W)$ and *c*_*n*_(*W*) are of the order $n^{b}=n^{(1-2\alpha )/(1+2\alpha )}$, almost surely. This follows because $D_{1,n}^{L}(c,W)$ behaves almost surely as its mean $\mathord \mathrm {E} D_{1,n}^{L}(c,W)$, which is of the order *n*(*cn*)^−*α*^. This was shown for the risk-based function $D_{1,n}^{R}$ in the preceding section, and extends to the likelihood-based function.

## Counterexample

Theorem 1 shows that the credible band will cover a function that is Hölder of order at least equal to its order of self-similarity. If the two measures of smoothness do not match, then the empirical or hierarchical Bayes method to choose the scaling of the prior will choose a value that does not balance the square bias and variance, which can result in credible bands that are too narrow or too wide.

The two types of smoothness do not agree in general. For instance, for the Hölder exponent of the function *f*_*α*_(*t*) = |*x* − *t*|^*α*^, where *α* ∈ (0,1) and *x* is a fixed value in (1/2,1), is *α*. Below we show that it self-similar of order *β* = 1. In this section we give an example that is Hölder of order *α* ∈ (0,1), but for which the order of self-similarity is equal to 1/2. Thus the Hölder exponent can be both smaller or larger than the order of self-similarity. For *α* < 1/2 this will result in credible bands that are too narrow and hence give a mistaken impression of remaining uncertainty, while for *α* > 1/2 the credible bands will cover but be unnecessarily wide. Thus despite the positive results expressed in Theorems 1 and 2, credible bands are not necessarily accurate.

**Figure 1:**
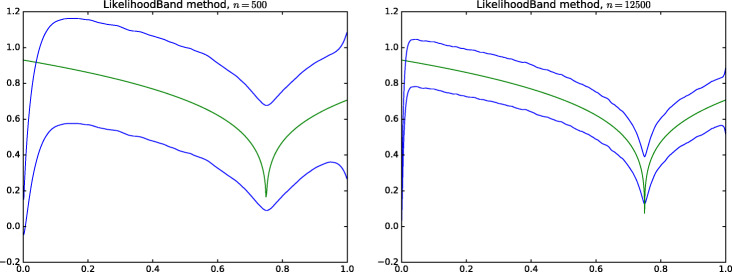
Credible band for the function *f*(*x*) = |*x* − 3/4|^1/4^, for *n* = 500 and *n* = 12500, for $\hat c_{n}$ set by the likelihood-based empirical Bayes method

We prove below that the Fourier coefficients of *f*_*α*_ satisfy *f*_*j*_ ≍ *j*^− 1^, for any *α* ∈ (0,1), which implies that it is self-similar of order $\beta = \frac 12$. The bias of the posterior mean at the point *x* has the exact order (*cn*)^−*α*/2^, by Corollary 3.1 in Sniekers and van der Vaart (2015b). The self-similarity suggests that $\tilde {c}_{n}$ and $\hat c_{n}$ will be of the order $\tilde {c}_{n}\asymp 1$. Then for *α* < 1/2 the bias $\sup _{x\in J_n}|\mu _{n}(x,\tilde {c}_{n})| \gtrsim n^{-a/2}$, which is much bigger than the width of the credible band $\sqrt {\log n} (\tilde {c}_{n}/n)^{1/4}$. Figure 1 illustrates this with a simulation for the true function with *α* = 1/4.

Since *f* ∈ *C*^*α*^, we have $|{\sum }_{j=n+1}^{\infty } f_{j} e_{j}(t)| \lesssim n^{-\alpha } \log n$ by Theorem 10.8 of Chapter 2 in Zygmund (1988). For *α* > 1/2 it can be shown that a *D*_1,*n*_(*c*,*f*) ≍ *n*(*cn*)^− 1/2^ on *I*_*n*_. From this it follows that the credible sets $\hat C_{n}(L)$ have asymptotic coverage one if $\alpha >\frac 12$, but the diameter is suboptimal; it is of the order $\sqrt {\log n} n^{-1/4}\gg (n/\log n)^{-\alpha /(1+2\alpha )}$.

We now prove that the Fourier coefficients of *f* are of the order *j*^− 1^. Consider

$$ \begin{array}{@{}rcl@{}} f_{j+1}=\sqrt{2}{{\int}_{0}^{1}} |x-t|^{\alpha}\sin(\pi r t) \text{d} t &= \frac{\sqrt{2}}{r} {{\int}_{0}^{r}} \left|x-\frac{s}{r}\right|^{\alpha} \sin(\pi s) \text{d} s, \end{array} $$

where *r* = *j* + 1/2. We may write

$$ {{\int}_{0}^{r}} \left|x-\frac{s}{r}\right|^{\alpha} \sin(\pi s) \text{d} s = \sum\limits_{k=0}^{2j} {\int}_{\frac{1}{2}k}^{\frac{1}{2}(k+1)} \left|x-\frac{s}{r}\right|^{\alpha} \sin(\pi s) \text{d} s=:\sum\limits_{k=0}^{2j} I_{k}. $$

We prove that for *r* sufficiently large this sum is bounded from above and below by a positive constant. Note that if *k* mod 4 is either 0 or 1, *I*_*k*_ is positive, otherwise it is negative. In the first case and for *k* + 1 < 2*xr*, we have

$$ \left( x-\frac{k+1}{2r}\right)^{\alpha}\cdot \frac{1}{\pi}\leq I_{k} \leq \left( x-\frac{k}{2r}\right)^{\alpha}\cdot \frac{1}{\pi}. $$

In the second case and for *k* + 1 < 2*xr*, we have

$$ -\left( x-\frac{k}{2r}\right)^{\alpha}\cdot \frac{1}{\pi}\leq I_{k} \leq-\left( x-\frac{k+1}{2r}\right)^{\alpha}\cdot \frac{1}{\pi}. $$

Similarly, in the first case and for *k* > 2*xr*, we have

$$ \left( \frac{k}{2r}-x\right)^{\alpha}\cdot \frac{1}{\pi}\leq I_{k} \leq\left( \frac{k+1}{2r}-x\right)^{\alpha}\cdot \frac{1}{\pi}. $$

Finally, in the second case and for *k* > 2*xr*, we have

$$ -\left( \frac{k+1}{2r}-x\right)^{\alpha}\cdot \frac{1}{\pi}\leq I_{k} \leq -\left( \frac{k}{2r}-x\right)^{\alpha}\cdot \frac{1}{\pi}. $$

Setting *k*_0_ := ⌊2*xr*⌋, *m*_0_ = *k*_0_ − 5 − (*k*_0_ mod 4) and *m*_1_ = *k*_0_ + 5 − (*k*_0_ mod 4) + 2(*j* mod 2), we may write

$$ \sum\limits_{k=0}^{2j} I_{k} = \sum\limits_{k=0}^{m_{0}} I_{k} + \sum\limits_{k=m_{1}}^{2j} I_{k} + o(1). $$

Note that

$$ \sum\limits_{k=0}^{m_{0}} I_{k} = \sum\limits_{k=0}^{(m_{0}-3)/4} \left[(I_{4k} + I_{4k+2}) + (I_{4k+1}+I_{4k+3})\right]. $$

Set $\tilde {m}_{0}=(m_{0}-3)/4$. Applying the mean value theorem, we see that for *b* > *a* we have

$$ \begin{array}{@{}rcl@{}} {\sum}_{k=0}^{\tilde{m}_{0}} &&{}\left( \left( x-\frac{a+4k}{2r}\right)^{\alpha}-\left( x-\frac{b+4k}{2r}\right)^{\alpha}\right) = \alpha{\sum}_{k=0}^{\tilde{m}_{0}}\left( \xi_{k}\right)^{\alpha-1} \frac{b-a}{2r}\\ &\leq& \frac{\alpha(b-a)}{2r}\sum\limits_{k=0}^{\tilde{m}_{0}}\left( x-\frac{b+4k}{2r}\right)^{\alpha-1} \leq \frac{\alpha(b-a)}{2r} {\int}_{0}^{\tilde{m}_{0}+1}\left( x-\frac{b+4k}{2r}\right)^{\alpha-1} \text{d} k\\ &=&-\frac{\alpha(b-a)}{4\alpha}\left[\left( x-\frac{b+4(\tilde{m}_{0}+1)}{2r}\right)^{\alpha} - \left( x-\frac{b}{2r}\right)^{\alpha}\right]\to \frac{(b-a)}{4}x^{\alpha}. \end{array} $$

Here we use *ξ*_*j*_ ≥ *x* − (*b* + 4*k*)/(2*r*), but we can apply the same argument with *ξ*_*j*_ ≤ *x* − (*a* + 4*k*)/(2*r*) to obtain a lower bound (changing the upper limit of the integral to $\tilde {m}_{0}$), which is asymptotically the same as the upper bound. Using this, we can bound $ {\sum }_{k=0}^{\tilde {m}_{0}} I_{k}$ from above by

$$ \begin{array}{@{}rcl@{}} && \frac{1}{\pi} \sum\limits_{k=0}^{\tilde{m}_{0}} \left( x-\frac{4k}{2r}\right)^{\alpha}-\left( x-\frac{3+4k}{2r}\right)^{\alpha}+\left( x-\frac{1+4k}{2r}\right)^{\alpha}-\left( x-\frac{4+4k}{2r}\right)^{\alpha}\\ &&\quad\leq -\frac{3}{4\pi}\!\left[\left( \!x-\frac{7+4\tilde{m}_{0}}{2r}\right)^{\alpha}\!- \left( \!x-\frac{3}{2r}\right)^{\alpha} \!+ \left( \!x-\frac{8+4\tilde{m}_{0}}{2r}\right)^{\alpha}\! - \left( \!x-\frac{4}{2r}\right)^{\alpha}\right] \rightarrow\frac{3}{2\pi}x^{\alpha}, \end{array} $$

and from below by

$$ \begin{array}{@{}rcl@{}} && \frac{1}{\pi} \sum\limits_{k=0}^{\tilde{m}_{0}} \left( \!x-\frac{1+4k}{2r}\right)^{\alpha}\!\!-\left( \!x-\frac{2+4k}{2r}\right)^{\alpha}\!\!+\left( \!x-\frac{2+4k}{2r}\right)^{\alpha}\!\!-\left( \!x-\frac{3+4k}{2r}\right)^{\alpha}\\ &&\quad\geq -\frac{1}{4\pi}\left[\left( \!x - \frac{1+4\tilde{m}_{0}}{2r}\right)^{\alpha}\! - \left( \!x-\frac{1}{2r}\right)^{\alpha}\! + \left( \!x-\frac{2+4\tilde{m}_{0}}{2r}\right)^{\alpha}\! - \left( \!x-\frac{2}{2r}\right)^{\alpha}\right] \rightarrow \frac{1}{2\pi}x^{\alpha}. \end{array} $$

For the other sum we have

$$ \sum\limits_{k=m_{1}}^{2j} I_{k} =\sum\limits_{k = 0}^{\tilde{m}_{1}} \left[ (I_{2j-4k} + I_{2j-4k-2})+(I_{2j-4k-1} + I_{2j-4k-3})\right], $$

where $\tilde {m}_{1}=(2j-3-m_{1})/4$. Applying the mean value theorem again, we see that for *d* > *c* we have

$$ \begin{array}{@{}rcl@{}} &&\sum\limits_{k=0}^{m} \left( \left( \frac{2j-4k-c}{2r}-x\right)^{\alpha}-\left( \frac{2j-4k-d}{2r}-x\right)^{\alpha}\right) = \alpha{\sum}_{k=0}^{m}\left( \xi_{k}\right)^{\alpha-1} \frac{d-c}{2r}\\ &\leq& \frac{\alpha(d-c)}{2r}{\sum}_{k=0}^{m}\left( \frac{2j-4k-d}{2r}-x\right)^{\alpha-1} \leq \frac{\alpha(d-c)}{2r} {\int}_{0}^{m+1}\left( \frac{2j-4k-d}{2r}-x\right)^{\alpha-1} \text{d} k\\ &=&-\frac{(d-c)}{4}\left[\left( \frac{2j-4(m+1)-d}{2r}-x\right)^{\alpha} - \left( \frac{2j-d}{2r}-x\right)^{\alpha}\right] \rightarrow \frac{(d-c)}{4}(1-x)^{\alpha}. \end{array} $$

Here we use *ξ*_*j*_ ≥ (2*j* − 4*k* − *d*)/(2*r*) − *x*, but applying the same argument with *ξ*_*j*_ ≤ (2*j* − 4*k* − *c*)/(2*r*) − *x*, we obtain a lower bound (changing the upper limit of the integral to $\tilde {m}_{1}$) that is asymptotically the same as the upper bound.

Proceeding as above and treating the cases *j* even and *j* odd separately, we obtain a lower bound for ${\sum }_{k=m_{1}}^{2j} I_{k}$ that converges to − (1 − *x*)^*α*^/(2*π*) and an upper bound that converges to (1 − *x*)^*α*^/(2*π*). Since *x* > 1/2, we have *x*^*α*^ > (1 − *x*)^*α*^, and the result follows.

By further subdividing each of the 2*j* parts, we can obtain better upper and lower bounds for the integral. We believe that it is possible to obtain a more accurate result this way and that the Fourier coefficients can be shown to satisfy $f_{j}\sim (\sqrt 2/\pi ) x^{\alpha }/(j+1/2)$.

## Posterior mean

The following proposition generalizes Theorem 2.2 of Sniekers and van der Vaart (2015b). It is proved by the same arguments.

### Proposition 15.

Let $i_{n}(x)=\max \limits \{i: x_{i,n}<x\}$ and $\lambda _{+}=1 + \sqrt {c/n_{+}}$$\sqrt {1+c/(4n_{+})}+\frac {1}{2} c/n_{+}$, and fix an arbitrary sequence *η*_*n*_ *↓* 0. The coefficients *a*_*i*,*n*_(*c*,*x*) satisfy, uniformly in *c* and *x* such that *c*/*n* ≤ *η*_*n*_ and $\left (x\wedge (1-x)\right )\sqrt {cn}\ge 1/\eta _{n}$,

8.1 $$   a_{i,n} (x,c) \sim\frac 12 \sqrt{\frac{c}{n}}\left\{\begin{array}{ll} \lambda_{+}^{-i_{n}(x)} \left[\lambda_{+}^{i} - \lambda_{+}^{-i}\right], & \text{ for } i\leq i_{n}(x), \\ \lambda_{+}^{i_{n}(x)} \left[\lambda_{+}^{-i+1} + \lambda_{+}^{-2n+ i}\right],& \text{ for } i > i_{n}(x). \end{array}\right. $$

Furthermore, the coefficients *a*_*i*,*n*_(*x*,*c*) are nonnegative, and for every *x* ∈ (1/*n*_+_,1] bounded by thrice the expression on the right side, which is in turn bounded above by $4\sqrt {c/n} \l _{+}^{-|i-i_{n}(x)|}$, uniformly in *c*/*n* ≤ *η*_*n*_. Moreover, for *x* ≥ 1/*n*_+_,

8.2 $$ \begin{array}{@{}rcl@{}} 1-\sum\limits_{i=1}^{n} a_{i,n}(x,c) = \frac {n_{+}}c a_{1,n}(x,c). \end{array} $$

### Proof 7.

Abbreviate *a*_*i*,*n*_(*x*,*c*) to *a*_*i*,*n*_ and *i*_*n*_(*x*) to *i*_*n*_. Since $\mathbf {Y_{n}^{T}}\mathbf a_{n}$ is the projection of $\sqrt c W_{x}$ onto the observations *Y*_*i*,*n*_, the coefficients *a*_*i*,*n*_ satisfy the orthogonality relations, for *i* = 1,…,*n*,

$$ 0=\mathord\mathrm{E}\left[\left( \sqrt{c} W_{x}- \mathbf{Y}_{n}^{T} \mathbf{a}_{n}\right) Y_{i,n}\right] =c \mathord\mathrm{E}\left[\left( W_{x}-\mathbf{W}_{n}^{T} \mathbf{a}_{n}\right) W_{x_{i,n}}\right]-a_{i,n}. $$

Evaluating this with *i* = 1 and noting that $\mathord \mathrm {E} W_{x}W_{x_{1,n}}=1/n_{+}$, for every *x* ≥ *x*_1,*n*_, which includes *x* = *x*_*i*,*n*_ for every *i*, we readily find Eq. 8.2.

The *n* projection equations can be written as a linear system with rows *i* = 1,…,*n*. Replacing first for *i* = 2,…,*n* the *i* th equation by the difference of the *i* th and (*i* − 1)th equations, and next replacing in the resulting system for *i* = 1,…,*n* − 1 the *i* th equation by the difference of the *i* th and (*i* + 1)th equations, we obtain the simplified linear system

8.3 $$  \left( \begin{array}{lllll} 2+{c}/{n_{+}} & -1 & 0 & {\cdots} & 0\\ -1 & 2+{c}/{n_{+}} & -1 & {\cdots} & 0\\ 0 & -1 & {\ddots} & & {\vdots} \\ {\vdots} & {\vdots} & &2+{c}/{n_{+}} & -1 \\ 0 & 0 & {\cdots} & -1 & 1+{c}/{n_{+}} \end{array}\right) \left( \begin{array}{lllll} a_{1,n} \\ a_{2,n} \\ \vdots\\ \phantom{\vdots}\\ {\vdots} \\ a_{n,n} \end{array}\right) =c \left( \begin{array}{lllll} 0 \\ {\vdots} \\ 0 \\ x_{i_{n}+1,n}-x \\ x-x_{i_{n},n} \\0\\ \vdots\\ 0 \end{array}\right). $$

The equations in rows 2,3,…,*i*_*n*_ − 1 and *i*_*n*_ + 2,…,*n* − 1 yield the recurrence relation, for *i* ∈{3,…,*i*_*n*_} and *i* ∈{*i*_*n*_ + 3,…,*n*},

8.4 $$ a_{i,n} =\left( 2+{c}/{n_{+}}\right)a_{i-1,n} -a_{i-2,n}. $$

This has characteristic polynomial $\lambda ^{2}-\left (2+{c}/{n_{+}}\right )\lambda +1$. The roots ł_+_ and ł_−_ of the corresponding characteristic equation possess product ł_+_ł_−_ = 1 and sum ł_+_ + ł_−_ = 2 + *c*/*n*_+_.

For *i* ∈{1,…,*i*_*n*_} the general solution takes the form $a_{i,n} =A\lambda _{+}^{i} + B\lambda _{-}^{i}$, for two constants *A* and *B*. The first equation in the system Eq. 8.3 is (ł_+_ + ł_−_)*a*_1,*n*_ − *a*_2,*n*_ = 0. Substituting the general solutions *a*_1,*n*_ = *A*ł_+_ + *B*ł_−_ and $a_{2,n}=A\l _{+}^{2}+B\l _{-}^{2}$ in this equation readily gives that *A* = −*B*. Hence

8.5 $$  a_{i,n} =A(\lambda_{+}^{i} -\lambda_{-}^{i}),\qquad i\in\{1,\ldots, i_{n}\}. $$

By Eq. 8.4 the numbers *b*_*i*,*n*_ = *a*_*n*−*i*+ 1,*n*_ satisfy the same recurrence relation $b_{i,n}=\left (2+{c}/{n_{+}}\right )b_{i-1,n} -b_{i-2,n}$, for *i* ∈{3,…,*n* − *i*_*n*_}, and hence $b_{i,n}= \tilde {A}\lambda _{+}^{i} +\tilde {B}\lambda _{-}^{i}$, for two constants $\tilde A$ and $\tilde B$ and every *i* ∈{1,…,*n* − *i*_*n*_}. The last row of the system of Eqs. 8.3 gives − *b*_2,*n*_ + (ł_+_ + ł_−_− 1)*b*_1,*n*_ = 0. Substituting the general solutions for *b*_2,*n*_ and *b*_1,*n*_ into this equation readily gives that $\tilde B=\l _{+}\tilde A=\tilde A/\l _{-}$. Translating back from *b*_*i*,*n*_ to *a*_*i*,*n*_, we conclude that

8.6 $$  a_{i,n} =\tilde A(\lambda_{+}^{n-i+1} +\lambda_{-}^{n-i}),\qquad i\in\{i_{n}+1,\ldots, n\}. $$

The *i*_*n*_th and (*i*_*n*_ + 1)th equations of Eq. 8.3 can be written

8.7 $$ \begin{array}{@{}rcl@{}} &&-a_{i_{n}-1,n} +(\l_{+}+\l_{-}) a_{i_{n},n} - a_{i_{n}+1,n} =c(x_{i_{n}+1,n}-x), \end{array} $$

8.8 $$ \begin{array}{@{}rcl@{}} &&-a_{i_{n},n} +(\l_{+}+\l_{-}) a_{i_{n}+1,n} - a_{i_{n}+2,n} =c(x-x_{i_{n},n}). \end{array} $$

We substitute the general solutions Eqs. 8.5 and 48.6 to find, after simplification, that the constants *A* and $\tilde A$ are the solutions of the linear system

$$ \begin{array}{@{}rcl@{}} A(\lambda_{+}^{i_{n}+1} -\lambda_{-}^{i_{n}+1})-\tilde A(\lambda_{+}^{n-i_{n}} +\lambda_{-}^{n-i_{n}-1})&=&c(x_{i_{n}+1,n}-x) ,\\ -A(\lambda_{+}^{i_{n}} -\lambda_{-}^{i_{n}})+\tilde A(\lambda_{+}^{n-i_{n}+1} +\lambda_{-}^{n-i_{n}})&=&c(x-x_{i_{n},n}). \end{array} $$

The determinant of this linear system can be calculated to be ${\Delta }_{n}=\l _{+}^{n}(\l _{+}^{2}-1)-\l _{-}^{n-1}(\l _{-}^{2}-1)=\l _{+}^{n}(\l _{+}^{2}-1)(1+\l _{+}^{-2n-1})$. Then

8.9 $$  \left( \begin{array}{ll} \l_{+}^{i_{n}} A\\ \l_{+}^{n-i_{n}}\tilde A\end{array}\right) =\frac {c\l_{+}^{n}} {{\Delta}_{n}} \left( \begin{array}{ll} \l_{+} (1+\l_{+}^{-2(n-i_{n})-1})&1+\l_{+}^{-2(n-i_{n})+1}\\ 1-\l_{+}^{-2i_{n}}&\l_{+}(1-\l_{+}^{-2i_{n}-2}) \end{array}\right) \left( \begin{array}{ll} x_{i_{n}+1,n}-x\\ x-x_{i_{n},n}\end{array}\right). $$

Here $\l _{+}-1\sim \sqrt {c/n}\rightarrow 0$ and $\l _{+}^{2}-1\sim 2\sqrt {c/n}$ uniformly in $c/n\rightarrow 0$, so that $c\l _{+}^{n}/{\Delta }_{n}\sim \sqrt {cn}/2$. The four entries of the matrix are all smaller than $1+\l _{+}\sim 2$ uniformly in $c/n\rightarrow 0$. The coordinates of the vector on the far right are nonnegative and add up to 1/*n*_+_, as $x_{i_{n},n}<x\le x_{i_{n}+1,n}$, by the definition of *i*_*n*_. Together with Eqs. 8.5 and 8.6 this shows that thrice the expression on the right side of Eq. 8.1 is an upper bound on *a*_*i*,*n*_(*x*,*c*).

Since *i*_*n*_ < *xn*_+_ ≤ *i*_*n*_ + 1, we have that $\left (i_{n}\wedge (n-i_{n})\right )\sqrt {c/n}\rightarrow \infty $, uniformly in *x* and *c* such that $\left (x\wedge (1-x)\right )\sqrt {cn}\ge l_{n}\rightarrow \infty $ and *c* ≤ *n*. Since $\log \l _{+}^{k}\sim k\sqrt {c/n}$, this shows that the 2 × 2 matrix in Eq. 8.9 converges to the matrix with all four entries equal to 1 uniformly in the same set of *x* and *c*. This gives the asymptotic equivalence Eq. 8.1. □

## Proof of Lemma 4

### Proof 8 (Proof of Eq. 2.2).

By Proposition 15 every coefficient *a*_*i*,*n*_(*x*,*c*) is nonnegative and bounded above by $6\sqrt {c/n} \l _{+}^{-|i_{n}-i|}$. Here $\l _{+}=1+\sqrt {c/n}+O(c/n)$ is bounded below by $e^{\sqrt {c/n}/2}$ uniformly in $c/n\le \eta _{n}\rightarrow 0$, since 1 + 3*x*/4 ≥ *e*^*x*/2^ for *x* ∈ [0,1], and hence $\l _{+}^{-|i_{n}-i|}\le e^{-|i_{n}-i|\sqrt {c/n}/2}$. Also $|i_{n}/n-x|\sqrt {cn}\le \sqrt {c/n}\rightarrow 0$, uniformly for $c/n\le \eta _{n}\rightarrow 0$, so that $e^{(i_{n}/n-x)\sqrt {cn}}$ is bounded. □

### Proof 9 (Proof of Eq. 2.1).

By Proposition 15, for *i* ≤ *i*_*n*_(*x*),

$$ \begin{array}{@{}rcl@{}} \log \left[\frac{2n a_{i,n}(x,c)}{\sqrt{cn} e^{-(x-x_{i,n})\sqrt{cn}} }\right] =-(i_{n}-i)\log \l_{+}+\sqrt{cn}(x-x_{i,n}) +\log (1-\l_{+}^{-2i})+o(1). \end{array} $$

Here $\log \l _{+}=\sqrt {c/n}+O(c/n)$, uniformly in $c/n\le \eta _{n}\rightarrow 0$, so that the first term is equivalent to $-(x_{i_{n},n}-x_{i,n})\sqrt {cn}$ up to a remainder of order $|x_{i_{n},n}-x_{i,n}|c$, which tends to zero uniformly in |*x* − *x*_*i*,*n*_|*c* ≤ *η*_*n*_, since $|x_{i_{n},n}-x|c\le 2c/n$. The approximation $-(x_{i_{n},n}-x_{i,n})\sqrt {cn}$ combines with the second term on the right side of the display to $-(x_{i_{n},n}-x)\sqrt {cn}$, which is of the order $\sqrt {c/n}\le \sqrt {\eta _{n}}\rightarrow 0$. For $x\sqrt {cn}\ge \eta _{n}^{-1}$ and $|x_{i,n}-x|\sqrt {cn}<(1/2)\eta _{n}^{-1}$, we have $i\sqrt {c/n}\rightarrow \infty $, whence $\l _{+}^{-2i} \rightarrow 0$, and the last term on the right tends to zero. For *i* > *i*_*n*_(*x*) the proof is similar. □

### Proof 10 (Proof of Eq. 2.3).

By Proposition 15, uniformly in *x* ≥ 1/*n*_+_ and $c/n\le \eta _{n}\rightarrow 0$,

$$1-\sum\limits_{i=1}^n a_{i,n}(x,c) =\frac {n_{+}}c a_{1,n}(x,c)\lesssim \sqrt{\frac n{c}} \l_{+}^{-i_{n}} (\l_{+}-\l_{+}^{-1})\sim \l_{+}^{-i_{n}}\left( 1+O(\sqrt{c/n})\right).$$

The logarithm of this is bounded above by $-i_{n}\log \l _{+}\sim -i_{n}\sqrt {c/n}\le -(x-1/n_{+})\sqrt {cn}/2\le -x\sqrt {cn}/2+o(1)$. □

### Proof 11 (Proof of Eq. 2.4).

If $\left (x\wedge (1-x)\right )\sqrt {cn}\ge 2\log n$, then $i_{n}\wedge (n-i_{n})\ge 2\log n\sqrt {n/c}(1-o(1))$, uniformly in *c* ≤ *n*. Thus we can choose *j*_*n*_ with $(3/2)\log n\sqrt {n/c}\le j_{n}\le (7/4)i_{n}\wedge (n-i_{n})$, and decompose ${\sum }_{i=1}^n a_{i}(x,c)(x_{i,n}-x)$ as

9.1 $$ \begin{array}{@{}rcl@{}} &&\sum\limits_{j=1}^{j_{n}} \left[a_{i_{n}-j+1,n}(x,c)(x-x_{i_{n}-j+1,n})+a_{i_{n}+j,n}(x,c)(x-x_{i_{n}+j,n})\right]\\ &+&\underset{text{ or } i>i_{n}+j_{n}}{\sum\limits\limits_{i\le i_{n}-j_{n}}} a_{i,n}(x,c)(x-x_{i,n}). \end{array} $$

For *x* > 1/*n*_+_ and $c/n\le \eta _{n}\rightarrow 0$, the second term is bounded above by a multiple of

$$ \sqrt{\frac cn}\sum\limits_{i\le i_{n}-j_{n}}\l_{+}^{-(i_{n}-i)} +\sqrt{\frac cn}\sum\limits_{i>i_{n}+j_{n}}2\l_{+}^{-(i-i_{n})} \lesssim \sqrt{\frac cn}\frac{\l_{+}^{-j_{n}}}{1-\l_{+}^{-1}}. $$

Since $1-\l _{+}^{-1}\sim \sqrt {c/n}$, uniformly in $c/n\le \eta _{n}\rightarrow 0$, this is bounded above by a multiple of 1/*n* if $j_{n}\log \l _{+}\ge \log n$. Since $\log \l _{+}\sim \sqrt {c/n}$, uniformly in $c/n\le \eta _{n}\rightarrow 0$, this is certainly the case under the assumption that $j_{n}\ge 3/2\log n\sqrt {n/c}$. To bound the first term of Eq. 9.1, we first note that for $i_{n}\wedge (n-i_{n})\gg \sqrt {n/c}$, which we have assumed, the four entries in the 2 × 2 matrix in Eq. 8.9 are $1+O(\sqrt {c/n})$, so that the quotient of the two coordinates of the vector on the left is $1+O(\sqrt {c/n})$ as well, uniformly in $c/n\le \eta _{n}\rightarrow 0$ and $\left (x\wedge (1-x)\right )\sqrt {cn}\ge 2\log n$. Combining this with Eq. 8.9 and Eq. 8.6, we see

$$ \begin{array}{@{}rcl@{}} \frac{a_{i_{n}-j+1,n}}{a_{i_{n}+j,n}}=\frac A{\tilde A}\frac{\l_{+}^{i_{n}-j+1}-\l_{-}^{i_{n}-j+1}}{\l_{+}^{n-i_{n}-j+1}+\l_{-}^{n-i_{n}-j}} =\l_{+}^{n-2i_{n}}\left[1+O\left( \sqrt{\frac cn}\right)\right] \frac{\l_{+}^{i_{n}-j+1}(1-\l_{+}^{-2i_{n}+2j-2})}{\l_{+}^{n-i_{n}-j+1}(1+\l_{+}^{-2n+2i_{n}+2j-1})}. \end{array} $$

The powers of ł_+_ cancel and $1-\l _{+}^{-2i_{n}+2j-2}$ and $1+\l _{+}^{-2n+2i_{n}+2j-1}$ are $1+O(\sqrt {c/n})$ for *j* ≤ *j*_*n*_, since $\left ((i_{n}-j_{n})\wedge (n-i_{n}-j_{n})\right )\ge (1/4)\log n\sqrt {n/c}$. We conclude that $a_{i_{n}-j+1,n}=a_{i_{n}+j,n}(1+R_{j,n})$, for a remainder satisfying $|R_{j,n}|\lesssim \sqrt {c/n}$, so that the first term in Eq. 9.1 is bounded above by

$$ \begin{array}{@{}rcl@{}} &&\sum\limits_{j=1}^{j_{n}} \left[a_{i_{n}+j,n}(x,c) \left( 2x-x_{i_{n}-j+1,n}-x_{i_{n}+j,n})+(x-x_{i_{n}-j+1,n})R_{j,n}\right)\right]\\ &&\lesssim \frac 1n\sum\limits_{j=1}^{j_{n}} a_{i_{n}+j,n}(x,c)+\sum\limits_{j=1}^{j_{n}} \frac cn\l_{+}^{-j+1}\frac j n. \end{array} $$

The first term is bounded by 1/*n* as the sum of the coefficients is bounded by 1. The second term is bounded above by a multiple of $(c/n^{2}){\sum }_{j=0}^{\infty } (j\l _{+}^{-j})$ and is also bounded by a multiple of 1/*n*, uniformly in $c/n\le \eta _{n}\rightarrow 0$. □

### Proof 12 (Proof of Eq. 2.5).

For given *x* and $M_{n}\rightarrow \infty $ we have, by Eq. 2.2,

$$ \sum\limits_{i: |x_{i,n}-x|>M_{n}/\sqrt{cn}} a_{i,n}^{2}(x,c) \lesssim \frac 1n\sqrt{cn} e^{-M_{n}/2}\sum\limits_{i=1}^n a_{i,n}(x,c)\le \frac 1n\sqrt{cn} e^{-M_{n}/2}. $$

For $M_{n}\rightarrow \infty $ this is of smaller order than $\sqrt {c/n}$. On the other hand, if $c/n\le \eta _{n}\rightarrow 0$ and $M_{n}=1/\sqrt {\eta _{n}}$, then $M_{n}/\sqrt {cn}\le \sqrt {\eta _{n}}/c$ and hence by Eq. 15 (applied with $\sqrt {\eta _{n}}$ in the role of 2*η*_*n*_), uniformly in $\left (x\wedge (1-x)\right )\sqrt {cn}\ge 1/\eta _{n}\ge 1/\sqrt {\eta _{n}}$,

$$ \begin{array}{@{}rcl@{}}\sum\limits_{i: |x_{i,n}-x|\le M_{n}/\sqrt{cn}} a_{i,n}^{2}(x,c) &\sim& \frac c{4n} \sum\limits_{i: |x_{i,n}-x|\le M_{n}/\sqrt{cn}} e^{-2|x-x_{i,n}|\sqrt{cn}}\\ &\sim& \frac c4{\int}_{x-M_{n}/\sqrt{cn}}^{x+M_{n}/\sqrt{cn}} e^{-2|x-s|\sqrt{cn}} ds = \frac14\sqrt{\frac cn}{\int}_{-M_{n}}^{M_{n}} e^{-2|s|} ds\sim \frac14\sqrt{\frac c{n}}. \end{array} $$

By Lemma 18 the error made in the second step by approximating the sum by the integral is smaller than (*c*/*n*) times the maximum value of the integrand, which is 1, which is indeed of smaller order than the right side of the display. □

### Proof 13 (Proof of Eq. 2.6).

For given *x*,*y* we have, by Eq. 2.2,

$$ \begin{array}{@{}rcl@{}}\sum\limits_{i} a_{i,n}(x,c) a_{i,n}(y,c) &\lesssim& \frac cn\sum\limits_{i=1}^n e^{-|x-x_{i,n}|\sqrt{cn}/2-|y-x_{i,n}|\sqrt {cn}/2}\\ &\lesssim& c{{\int}_{0}^{1}} e^{-|x-s|\sqrt{cn}/2-|y-s|\sqrt{cn}/2} ds + \frac cn\sup_{s} e^{-|x-s|\sqrt{cn}/2-|y-s|\sqrt{cn}/2}. \end{array} $$

The integral can be evaluated to be no bigger than $\left (3\sqrt {c/n}+c|x-y|\right )$$e^{-|x-y|\sqrt {cn}/2}$; the second term is negligible. □

### Proof 14 (Proof of Eq. 2.7).

The function $\bar \sigma _{n}(x,y,c)$ is the sum of *τ*_*n*_(*x*,*y*,*c*) and the covariance function of the process ${\sum }_{i=1}^n a_{i,n}(x,c)(W_{x}-W_{x_{i,n}})$. For $x=x_{i_{n}+1,n}$ the increments $W_{x}-W_{x_{i,n}}$ can be written as a sum (for *i* ≤ *i*_*n*_) or negative sum (for *i* > *i*_*n*_) of the increments $V_{j}=W_{x_{j,n}}-W_{x_{j-1,n}}$ over the grid points. Substituting these sums in ${\sum }_{i=1}^n a_{i,n}(x,c)(W_{x}-W_{x_{i,n}})$ and exchanging the order of the resulting double sums yields that this sum is equal to

$$ \begin{array}{@{}rcl@{}} \sum\limits_{i\le i_{n}}\left[a_{i,n}(x,c)\sum\limits_{i<j\le i_{n}+1}V_{j}\right] - \sum\limits_{i>i_{n}+1}\left[a_{i,n}(x,c)\sum\limits_{i_{n}+1<j\le i}V_{j}\right] = \sum\limits_{j=2}^{n} A_{j,n}(x,c)V_{j}, \end{array} $$

where

$$ A_{j,n}(x,c)= \left\{\begin{array}{ll}{\sum}_{i=1}^{j-1}a_{i,n}(x,c),&\text { if } j\le i_{n}(x)+1,\\ -{\sum}_{i=j}^{n}a_{i,n}(x,c),&\text { if } j> i_{n}(x)+1. \end{array}\right. $$

By the independence of the increments it follows that

$$ \begin{array}{@{}rcl@{}} \text{cov}\left( \sum\limits_{i=1}^n a_{i,n}(x,c)(W_{x}-W_{x_{i,n}}), \sum\limits_{i=1}^n a_{i,n}(y,c)(W_{y}-W_{x_{i,n}})\right) =\frac1n_{+}\sum\limits_{j=1}^n A_{j,n}(x,c) A_{j,n}(y,c). \end{array} $$

For *j* ≤ *i*_*n*_(*x*) + 1 we have, by Eq. 2.2,

$$ \begin{array}{@{}rcl@{}} A_{j,n}(x,c)\lesssim \sqrt{\frac cn}\sum\limits_{i=1}^{j-1}e^{-(x-x_{i,n})\sqrt{cn}/2}\le \sqrt{cn}{\int}_{0}^{x_{j,n}}e^{-(x-s)\sqrt{cn}/2} ds \le 2e^{-|x-x_{j,n}|\sqrt{cn}/2}. \end{array} $$

For *j* > *i*_*n*_(*x*) + 1 the same bound is valid. This bound is of the same form as the bound on *a*_*j*_(*x*,*c*), except for a factor $\sqrt {c/n}$. It follows by the same arguments as for the proof of Eq. 2.6 that $(c/n){\sum }_{j=1}^n A_{j,n}(x,c) A_{j,n}(y,c)$ is bounded as ${\sum }_{j=1}^n a_{j,n}(x,c)a_{j,n}(y,c)=\tau _{n}(x,y,c)$.

For *x* not equal to a grid point, the exact representation of ${\sum }_{i=1}^n a_{i,n}(x,c)$$(W_{x}-W_{x_{i,n}})$ in terms of the increments *V*_*j*_ is retained if the definitions of $V_{i_{n}+1}$ and $V_{i_{n}+2}$ are modified to $W_{x}-W_{x_{i_{n},n}}$ and $W_{x_{i_{n}+2,n}}-W_{x}$. The variances of these variables are also bounded above by a multiple of 1/*n*, and hence the preceding derivation goes through. □

### Proof 15 (Proof of Eq. 2.8).

The orthogonality of the residual $\sqrt c W_{x}-(\sqrt c \mathbf W_{n}+\mathbf \varepsilon _{n})^{T}\mathbf a_{n}$ and $(\sqrt c \mathbf W_{n}+\mathbf \varepsilon _{n})^{T}\mathbf a_{n}$ gives that $c U(x,\mathbf x_{n})=(c U_{n}+I)\mathbf a_{n}$, for *U*_*n*_ the covariance matrix of $\mathbf W_{n}$ and *U*(*x*,**x**_*n*_) the vector with coordinates $\text {cov}(W_{x}, W_{x_{i,n}})=x\wedge x_{i,n}$. Therefore $(c^{-1}I+U_{n}) \mathbf {a}_{n}(x,c) = U(x,\mathbf x_{n})$ is free of *c* and hence

$$ \begin{array}{@{}rcl@{}} \left\| \mathbf{a}_{n}(x,c) - \mathbf{a}_{n}(x,d) \right\| = \left\|\left[(c^{-1}I+U_{n})^{-1}(d^{-1}I + U_{n})-I\right] \mathbf{a}_{n}(x,d)\right\| \leq \kappa \left\| \mathbf{a}_{n}(x,d)\right\|, \end{array} $$

for *κ* the largest eigenvalue of the matrix (*c*^− 1^*I* + *U*_*n*_)^− 1^(*d*^− 1^*I* + *U*_*n*_) − *I*. The eigenvalues of this matrix are given by (*c* − *d*)/(*d*(1 + *cλ*_*j*,*n*_)), for *λ*_*j*,*n*_ ≍ *n*/*j*^2^ the eigenvalues of *U*_*n*_, whence *κ* ≤|*c* − *d*|/*d*. Since $\left \| \mathbf {a}_{n}(x,d)\right \|^{2}= \tau _{n}(x,x,d)\asymp \sqrt {d/n}$ by Eq. 2.5, we obtain the bound |*c* − *d*|/(*d*^3/4^*n*^1/4^) on the preceding display.

To complete this to a proof of Eq. 2.8 we combine this with a bound on $\left \| \mathbf {a}_{n}(x,c)-\mathbf {a}_{n}(y,c) \right \|$. Since (*cU*_*n*_ + *I*)**a**_*n*_ = *cU*(*x*,**x**_*n*_) and *U* is continuous, the coefficients *a*_*i*,*n*_ depend continuously on *x*. Furthermore, Eq. 8.3 shows that **a**_*n*_ is differentiable with respect to *x* in every interval (*x*_*i*,*n*_,*x*_*i*+ 1,*n*_], as *i*_*n*_(*x*) is constant in such an interval and *x* appears only in the right side of Eq. 8.3. Differentiating across Eq. 8.3 we see that the derivatives $\mathbf a_{n}^{\prime }$ satisfy the same equation, except that the vector on the far right must be replaced by its derivative, which has − 1 and 1 as its *i*_*n*_st and (*i*_*n*_ + 1)st coordinates and zeros elsewhere. The same analysis as in the proof of Proposition 15 shows that

$$ a_{i,n}^{\prime}= \left\{\begin{array}{ll} A_{1}(\l_{+}^{i}-\l_{-}^{i}),& i\in\{1,\ldots, i_{n}\},\\ \tilde A_{1}(\l_{+}^{n-i+1}+\l_{-}^{n-i}),& i\in\{i_{n}+1,\ldots,n\}, \end{array}\right. $$

where *A*_1_ and $\tilde A_{1}$ are constants satisfying the analogue of Eq. 8.9 given by

$$ \left( \begin{array}{ll} \l_{+}^{i_{n}} A_{1}\\ \l_{+}^{n-i_{n}}\tilde A_{1}\end{array}\right) =\frac {c\l_{+}^{n}} {{\Delta}_{n}} \left( \begin{array}{ll} \l_{+} (1+\l_{+}^{-2(n-i_{n})-1})&1+\l_{+}^{-2(n-i_{n})+1}\\ 1-\l_{+}^{-2i_{n}}&\l_{+}(1-\l_{+}^{-2i_{n}-2}) \end{array}\right) \left( \begin{array}{ll} -1\\ 1\end{array}\right). $$

As noted before, the four entries of the 2 × 2 matrix in the display tend to 1, uniformly in $\left (x\wedge (1-x)\right )\sqrt {cn}\rightarrow \infty $ and $c/n\rightarrow 0$. Since this matrix maps the vector (− 1,1)^*T*^ to 0, we expand the right side of the display more precisely as

$$ \frac {\sqrt{cn}}2\left( 1+o(1)\right) \left( \begin{array}{ll} \l_{+}+o(c/n) &1+o(c/n)\\ 1+o(c/n)&\l_{+}+o(c/n) \end{array}\right) \left( \begin{array}{ll} -1\\ 1\end{array}\right) \sim \frac{\sqrt{cn}}2(\l_{+}-1) \left( \begin{array}{ll} -1\\ 1\end{array}\right) \sim \frac c2 \left( \begin{array}{ll} -1\\ 1\end{array}\right). $$

We conclude that $A_{1}\sim -\sqrt {cn}A$ and $\tilde A_{1}\sim \sqrt {cn}\tilde A$, for *A* and $\tilde A$ given in the proof of Proposition 15, so that $|a_{i,n}^{\prime }|\sim \sqrt {cn} a_{i,n}$, for *x*∉{*x*_1,*n*_,…,*x*_*n*,*n*_}, where the sign is negative if *i* ≤ *i*_*n*_(*x*) and positive otherwise. Therefore, for *x* < *y*,

$$ \left\| \mathbf{a}_{n}(x,c)-\mathbf{a}_{n}(y,c) \right\|^{2} = \sum\limits_{i}\left( {{\int}_{x}^{y}}a_{i,n}^{\prime}(s) ds\right)^{2} \!\le cn |y-x|{{\int}_{x}^{y}}\sum\limits_{i} a_{i,n}^{2}(s,c) ds\le cn |y-x|^{2}\sqrt{\frac cn}, $$

since $ \|\mathbf a_{n}(x,c)\|^{2}\lesssim \sqrt {c/n}$, uniformly in its argument, in view of Eq. 2.5. □

### Proof 16 (Proof of Eq. 2.9).

The left side of the inequality is the second moment of the increment over [*x*,*y*] of the process with covariance function $\bar \sigma _{n}(x,y,c)$. By the representation as used in the proof of Eq. 2.7,

$$ \begin{array}{@{}rcl@{}} c \mathord\mathrm{E} \left[\sum\limits_{i=1}^n a_{i,n}(x,c)(W_{x}-W_{x_{i,n}})- \sum\limits_{i=1}^n a_{i,n}(y,c)(W_{y}-W_{x_{i,n}})\right]^{2} \sim\frac cn\sum\limits_{j=2}^{n} \left( A_{j,n}(x,c)- A_{j,n}(y,c)\right)^{2}. \end{array} $$

The functions *A*_*j*,*n*_ are continuous in *x*, and differentiable with respect to *x* except at grid points, with derivatives satisfying $A_{j,n}^{\prime }={\sum }_{i=1}^{j-1}a_{i,n}^{\prime }$, for *j* ≤ *i*_*n*_ and $A_{j,n}^{\prime }={\sum }_{i=j}^{n}a_{i,n}^{\prime }$, for *j* > *i*_*n*_. By the result of the preceding paragraph we have in both cases that $|A_{j,n}^{\prime }|\lesssim \sqrt {cn} A_{j,n}$. Therefore, by the same argument as in the preceding paragraph the preceding display is bounded above by $cn |y-x|^{2}\sup _{x} {\sum }_{j} A_{j,n}^{2}(x,c)$. By Eq. 2.7 applied with *x* = *y*, this is bounded by the right side of Eq. 2.9. □

## Proof of Lemma 7

The first part of the following proof is adapted from Example 34 and the last parts from Examples 22 and 23 in Sniekers and van der Vaart (2015a).

Since |*f*_*j*_|≤ *Mn*^− 1/2−*β*^, for every *j*, we have for *ℓ* ≥ 1,

$$ |f_{(2n+1)\ell+i}|\vee|f_{(2n+1)\ell+2n+2-i}|\le \frac {M}{n^{1/2+\beta}\ell^{1/2+\beta}}. $$

Since ${\sum }_{l\ge 1}l^{-1/2-\beta }=:C_{\beta }<\infty $, this shows that the series Eq. 3.2 that defines the aliased coefficients converges. Because the term for *l* = 0 of the series is *f*_*j*_ − *f*_2*n*+ 2−*j*_ and |*f*_2*n*+ 2−*j*_|≤ *M*(*n* + 2)^− 1/2−*β*^ for every *j* ≤ *n*, we see that the rescaled coefficients $\tilde f_{i,n}=f_{i,n}/\sqrt {n_{+}}$ satisfy $|\tilde f_{i,n}-f_{i}|\le 2C_{\beta } M n^{-1/2-\beta }$, so that $|\tilde f_{i,n}|\le 3C_{\beta } M i^{-1/2-\beta }$ and the left side of Eq. 3.3 satisfies

10.1 $$  \sum\limits_{i=m}^{n} \tilde f_{i,n}^{2} \le18C_{\beta}^{2} \frac {M^{2}} {m^{2\beta}}. $$

We wish to show that the right side of Eq. 3.3 is lower bounded by the expression on the right, where we may assume that *m* satisfies *ρm* ≤ *n*, because otherwise there is nothing to prove. First we note that

$$ |\tilde f_{i,n}^{2} - {f_{i}^{2}}| = |\tilde f_{i,n}-f_{i}| |\tilde f_{i,n} + f_{i}|\le \frac{2C_{\beta} M}{n^{1/2+\beta}}\left( 2|f_{i}|+\frac {2C_{\beta} M}{n^{1/2+\beta}}\right)\le \frac {8C_{\beta}^{2} M^{2}}{n^{1/2+\beta}i^{1/2+\beta}}. $$

It follows that, for *f* self-similar with constants (*ε*_1_,*ρ*_1_) and any *ρ* ≥ *ρ*_1_ and *ρm* ≤ *n*,

10.2 $$ \sum\limits_{i=m}^{\rho m\wedge n} \tilde f_{i,n}^{2} \ge\sum\limits_{i=m}^{\rho m} {f_{i}^{2}} - \frac{8C_{\beta}^{2} M^{2}}{n^{1/2+\beta}}\sum\limits_{i=m}^{\rho m} \frac{1}{i^{1/2+\beta}} \gtrsim \frac {M^{2}}{m^{2\beta}}\left( \varepsilon_{1}-8C_{\beta}^{2}\frac{\rho-1}{\rho^{1/2+\beta}}\right). $$

For sufficiently large *ρ* the constant on the right is positive. It follows that *f* is discretely self-similar with constants *M*, $\varepsilon =\varepsilon _{1}-8C_{\beta }^{2}(\rho -1)/\rho ^{1/2+\beta }$ and *ρ* ≥ *ρ*_1_ large enough that *ε* > 0. Combining this with Eq. 10.1 we also see that *f* satisfies the discrete polished tail condition Eq. 3.3 with constants $L=18C_{\beta }^{2}/\varepsilon $ and *ρ*.

Since 1 + *cλ*_*j*,*n*_ ≤ 1 + *ρ*^2^, for $j\geq \sqrt {cn}/\rho $ we have for discretely self-similar *f*,

$$ D_{1,n}^{R}(c,f) = \sum\limits_{i=1}^{n}\frac{f_{i,n}^{2}}{(1+c\lambda_{i,n})^{2}} \ge \frac 1{(1+\rho^{2})^{2}}\sum\limits_{i=\sqrt{cn}/\rho}^{n} f_{i,n}^{2} \gtrsim \frac{n\varepsilon M^{2}\rho^{2\beta}}{(1+\rho^{2})^{2}}\left( \frac{1}{cn}\right)^{\beta}. $$

For $D_{1,n}^{L}$ the same inequality is true, but with the factor (1 + *ρ*^2^)^2^ replaced by 1 + *ρ*^2^. Finally

10.3 $$ \begin{array}{@{}rcl@{}} \frac1{M^{2}}D_{1,n}^{R}(c,f)&\lesssim n \sum\limits_{j=1}^n \frac{ j^{-2\beta-1}}{(1+c\lambda_{j,n})^{2}} \lesssim n\sum\limits_{j=1}^n \frac{j^{3-2\beta}}{(j^{2}+cn)^{2}} \!\lesssim \left\{\begin{array}{lll} n(cn)^{-\beta} &\text{ if } \beta<2\\ n(cn)^{-2}\log (cn) &\text{ if } \beta=2\\ n(cn)^{-2}&\text{ if } \beta>2. \end{array}\right. \end{array} $$

The first case follows directly by Lemma 16, the second by writing

$$ n\sum\limits_{j=1}^n \frac{j^{3-2\beta}}{(j^{2}+cn)^{2}} = n\sum\limits_{j=1}^{\sqrt{cn}} \frac{j^{3-2\beta}}{(j^{2}+cn)^{2}} + n\sum\limits_{j=\sqrt{cn}+1}^{n} \frac{j^{3-2\beta}}{(j^{2}+cn)^{2}} $$

and applying a variant of the lemma to the second sum. The third case follows immediately by using *j*^2^ + *cn* > *cn*. For the likelihood-based method we have

10.4 $$ \begin{array}{@{}rcl@{}} \frac1{M^{2}}\! D_{1,n}^{L}(c,f)&\!\!\!\!\lesssim\! n\sum\limits_{j=1}^n \frac{ j^{-2\beta-1}}{1+c\lambda_{j,n}} \lesssim n\sum\limits_{j=1}^n \frac{j^{1-2\beta}}{j^{2}+cn} \!\lesssim\! \left\{{}\begin{array}{ll} n(cn)^{-\beta} &\text{ if } \beta<1\\ c^{-1}\log (cn) &\text{ if } \beta=1\\ c^{-1}&\text{ if } \beta>1. \end{array}\right. \end{array} $$

This concludes the proof of Lemma 7.

## Technical results for easy reference

For easy reference we state technical results from earlier papers.

### Lemma 16 (Sniekers and van der Vaart (2015a), Lemma 43).

Let *γ* > − 1, *m* ≥ 1 and $\nu \in \mathbb {R}$ such that *γ* − *mν* < − 1. Then

11.1 $$ \sum\limits_{j=1}^{n} \frac{j^{\gamma}}{(j^{m}+c n)^{\nu}} = C_{\gamma,\nu,m} (cn)^{\gamma/m-\nu+1/m}\left( 1+o(1)\right) $$

uniformly for *c* ∈ [*l*_*n*_/*n*,*n*^*m*− 1^/*l*_*n*_] as $n\rightarrow \infty $, for any $l_{n}\rightarrow \infty $. The constant is given by

$$ C_{\gamma,\nu,m} = {\int}_{0}^{\infty} \frac{u^{\gamma}}{(u^{m}+1)^{\nu}} \text{d} u. $$

Furthermore, the left side of (11.1) has the same order as the right side uniformly in *c* ∈ [*l*_*n*_/*n*,*n*^*m*− 1^], for any $l_{n}\rightarrow \infty $, possibly with a smaller constant.

### Lemma 17 (Sniekers and van der Vaart (2015a), Lemma 42).

Let $D_{1}: I_n \to (0,\infty )$ be a decreasing function and $D_{2}: I_n\to (0,\infty )$ an increasing function. Suppose that there exist $a,b,B,B^{\prime }>0$ such that

$$ \begin{array}{@{}rcl@{}} D_{1}(Kc)&\le& K^{-a}D_{1}(c),\qquad \text{ for any }\quad K>1,\\ B^{\prime} k^{b} D_{2}(c) \geq D_{2}(kc)&\ge& Bk^{b} D_{2}(c)\qquad \text{ for any }\quad k<1. \end{array} $$

Let $\tilde c$ satisfy $D_{1}(\tilde c)= D_{2}(\tilde c)$, and for a given constant *E* ≥ 1, define ${\Lambda }=\left \{c: (D_{1}+D_{2})(c)\le E (D_{1}+D_{2})(\tilde c)\right \}$. Then

(i) *D*_1_(*c*) ≤ *B*^− 1^(2*E*)^1+*b*/*a*^*D*_2_(*c*), for every *c* ∈Λ.
(ii) ${\Lambda }\subset \left [(2E)^{-1/a}\tilde c, (2EB^{\prime })^{1/b}\tilde c\right ]$.

### Lemma 18.

If $f: [0,1]\to \mathbb {R}_{\ge 0}$ is increasing on [0,*m*] and decreasing on [*m*,1], then ${{\int \limits }_{0}^{1}} f(x) dx-f(m)/n_{+}\le n_{+}^{-1}{\sum }_{i=1}^n f(i/n_{+})\le {{\int \limits }_{0}^{1}}f(x) dx +f(m)/n_{+}$.

### Proof 17.

This is elementary analysis. □
